# Ubiquitous Over-Expression of Chromatin Remodeling Factor *SRG3* Ameliorates the T Cell-Mediated Exacerbation of EAE by Modulating the Phenotypes of both Dendritic Cells and Macrophages

**DOI:** 10.1371/journal.pone.0132329

**Published:** 2015-07-06

**Authors:** Sung Won Lee, Hyun Jung Park, Sung Ho Jeon, Changjin Lee, Rho Hyun Seong, Se-Ho Park, Seokmann Hong

**Affiliations:** 1 Dept. of Bioscience and Biotechnology, Institute of Bioscience, Sejong University, Seoul 143–747, Korea; 2 Dept. of Life Science, Hallym University, Chuncheon 200–702, Korea; 3 Dept. of Biological Sciences, Institute of Molecular Biology and Genetics, Research Center for Functional Cellulomics, Seoul National University, Seoul 151–742, Korea; 4 School of Life Sciences and Biotechnology, Korea University, Seoul 136–701, Korea; Wayne State University, UNITED STATES

## Abstract

Although SWI3-related gene (SRG3), a chromatin remodeling factor, is critical for various biological processes including early embryogenesis and thymocyte development, it is unclear whether SRG3 is involved in the differentiation of CD4^+^ T cells, the key mediator of adaptive immune responses. Because it is known that experimental autoimmune encephalomyelitis (EAE) development is determined by the activation of CD4^+^ T helper cells, here, we investigated the role of SRG3 in EAE development using SRG3 transgenic mouse models exhibiting two distinct SRG3 expression patterns: SRG3 expression driven by either the CD2 or β-actin promoter. We found that the outcome of EAE development was completely different depending on the expression pattern of SRG3. The specific over-expression of SRG3 using the CD2 promoter facilitated EAE via the induction of Th1 and Th17 cells, whereas the ubiquitous over-expression of SRG3 using the β-actin promoter inhibited EAE by promoting Th2 differentiation and suppressing Th1 and Th17 differentiation. In addition, the ubiquitous over-expression of SRG3 polarized CD4^+^ T cell differentiation towards the Th2 phenotype by converting dendritic cells (DCs) or macrophages to Th2 types. SRG3 over-expression not only reduced pro-inflammatory cytokine production by DCs but also shifted macrophages from the inducible nitric oxide synthase (iNOS)-expressing M1 phenotype to the arginase-1-expressing M2 phenotype during EAE. In addition, Th2 differentiation in β-actin-SRG3 Tg mice during EAE was associated with an increase in the basophil and mast cell populations and in IL4 production. Furthermore, the increased frequency of Treg cells in the spinal cord of β-actin-SRG3 Tg mice might induce the suppression of and accelerate the recovery from EAE symptoms. Taken together, our results provide the first evidence supporting the development of a new therapeutic strategy for EAE involving the modulation of SRG3 expression to induce M2 and Th2 polarization, thereby inhibiting inflammatory immune responses.

## Introduction

The epigenetic machinery regulating the chromatin structure consists of DNA methylation, histone modification and ATP-dependent remodeling. Recent findings have contributed to the identification of the influence of epigenetic modifications on the development of various autoimmune diseases, including experimental autoimmune encephalomyelitis (EAE) [1]. SWI/SNF is one of the various families of ATP-dependent chromatin remodeling complexes, which regulate chromatin structure and DNA accessibility [2]. Among the diverse SWI/SNF complexes, SWI3-related gene (SRG3) is a core subunit that plays a vital role in the post-transcriptional stabilization of the major components of the SWI/SNF complex, including SNF5, BRG1, and BAF60a [3]. In addition to its essential roles in early embryogenesis, brain development [4] and extra-embryonic vascular development [5], SRG3 plays an important role in immunological functions such as the regulation of thymocyte development [6]. One previous study has shown that SWI/SNF complexes containing SRG3 and BRG1 induce the activation and proliferation of T cells via the regulation of AP-1 [7].

EAE is an animal model of human autoimmune diseases such as multiple sclerosis (MS), which is an inflammatory disease that leads to central nervous system (CNS) tissue damage, myelin sheath destruction and axonal loss or damage [8]. Innate immune cells including dendritic cells (DCs) and macrophages/microglia are antigen-presenting cells (APCs) that play central roles in the induction of EAE [9]. Particularly, it has been reported that DCs act as a key regulator of the induction of both immunity and tolerance to self-antigens [10]. Macrophages/microglia, known to act as effector cells mediating immune responses in EAE [9], can be classified into two phenotypes: the classical M1 phenotype, which displays a “pro-inflammatory” cytokine profile (e.g., TNFα, IL6, and inducible nitric oxide synthase (iNOS)), and the alternative M2 phenotype, which displays a “anti-inflammatory” cytokine profile (e.g., IL10 and arginase-1) [11]. M1 macrophages participate in inflammation and demyelination in the CNS during EAE, whereas M2 macrophages play essential roles in tissue repair and functional recovery [12]. EAE can be induced by immunization with self-antigens such as myelin proteins in various species, including mice, rats, guinea pigs and rabbits. It has been demonstrated that EAE is mediated by the activation of CD4^+^ T helper cells, particularly Th1 and Th17 cells [13].

Recently, one study demonstrated that the over-expression of SRG3 under the control of the human CD2 promoter exacerbated the development of EAE, suggesting that SRG3 expression in the T cell lineage significantly influences the pathogenesis of EAE [7]. CD4^+^ T helper cells have been implicated in regulating autoimmune responses. These cells consist of functionally and phenotypically heterogeneous subpopulations characterized by different cytokine profiles: IFNγ for Th1, IL4 for Th2, IL9 for Th9, IL17 for Th17, and TGFβ for Treg cells [14]. In EAE, Th1 cells play pathological roles in disease progression, whereas Th2 cells are associated with the recovery phase [15]. Previous studies have demonstrated that IL4 and Th2 cells play a vital role in the spontaneous remission of EAE in IL4 knockout mice in the PL/J genetic background [16], and IL4 gene delivery to the CNS using an adenoviral vector induced clinical and functional recovery from both progressive EAE and chronic progressive EAE [17]. Moreover, copolymer 1-induced Th2 cells accumulated in the CNS and protected against EAE induction via an anti-inflammatory effect [18]. In addition, it has been reported that Th17, Th9 and Treg cells are associated with clinical disease activity. Whereas Th17 and Th9 cells mediate EAE pathology [19], CD4^+^CD25^+^ Treg cells inhibit EAE development by directly inhibiting the expansion of pathogenic effector T cells [20, 21].

Recently, Jeong *et al*. demonstrated that SRG3 over-expression in the T cell lineage accelerated the development of MOG peptide-induced EAE in the C57BL/6 strain [7]. To dissect whether SRG3 over-expression affects CD4^+^ T cell differentiation, we introduced the SRG3 transgene by crossing SRG3 Tg mice with myelin basic protein (MBP)-specific TCR transgenic B10.PL mice [22]. For this purpose, we employed two types of SRG3 Tg mice exhibiting distinct cellular expression patterns: 1) SRG3 over-expression driven by the CD2 promoter (CD2-SRG3) and 2) SRG3 over-expression driven by the β-actin promoter (β-actin-SRG3). We found that the outcome of EAE development was completely different depending on the expression pattern of SRG3. The specific over-expression of SRG3 in T lineage cells facilitated EAE, whereas the ubiquitous over-expression of SRG3 in all cell types inhibited EAE. The ubiquitous over-expression of SRG3 polarized CD4^+^ T cell differentiation towards the Th2 phenotype by changing the phenotypes of DCs or macrophages. Here, our results suggest that SRG3 affects the polarization of CD4^+^ T cells by modulating the functions of APCs such as macrophages.

## Materials and Methods

### Mice and reagents

β-actin-SRG3 Tg C57BL/6 (B6) and CD2-SRG3 Tg B6 mice were backcrossed with MBP TCR Tg B10.PL mice more than eleven and seven times, respectively. MBP TCR Tg B10.PL mice [23] were provided from Dr. Luc Van Kaer (Vanderbilt University, Nashville, USA) by kind permission of Dr. Juan J. Lafaille (Rockefeller University, USA). The mice were bred and maintained at Sejong University and were 6–12 weeks old in the experiments performed, unless otherwise specified. The mice were fed a γ-irradiated sterile diet and autoclaved distilled water. The animal experiments were approved by the Institutional Animal Care and Use Committee at Sejong University (SJ-20100401009). TLR ligands were used as positive controls. Lipopolysaccharide (LPS) derived from Escherichia coli (serotype 0111:B4) was purchased from Sigma-Aldrich (St. Louis, MO, USA). Recombinant murine IL3 was purchased from R&D systems (Minneapolis, MN, USA).

### Genotyping of mice

To confirm the integration of the transgene, genomic DNA from tail biopsies was used to amplify an 800 bp fragment that was detectable only in mice carrying the SRG3 transgene. The primers used for the PCR genotyping were as follows: forward 5’-GAC TAG ACC AAA CAT CTA CCT C-3’; reverse 5’- GTC AAC TGA GCG ACT GGA TC-3’.

### Cell isolation and culture

Splenic CD4^+^ T cells were isolated using a magnetic activated cell sorting (MACS) system (Miltenyi Biotec, Germany) according to the manufacturer’s instructions. The CD4^+^ T cell population was >97% of all cells after MACS. Primary cells were cultured in complete RPMI medium consisting of RPMI 1640 (Gibco BRL, Gaithersburg, Maryland, USA) medium supplemented with 10% FBS, 10 mM HEPES, 2 mM L-glutamine, 100 units/ml penicillin-streptomycin, and 5 mM 2-mercaptoethanol.

### Flow cytometric analysis and gating strategy

The following mAbs from BD Biosciences were used: fluorescein isothiocyanate (FITC)-, phycoerythrin (PE)-Cy7- or allophycocyanin (APC)-conjugated anti-CD3ε (clone 145-2C11); FITC- or PE-Cy7-conjugated anti-CD4 (clone RM4-5); FITC- or APC-conjugated anti-CD11c (clone HL3); PE-Cy7-conjugated anti-CD11b (clone M1/70); PE-conjugated anti-Siglec-F (clone E50-2440); PE- or APC-conjugated anti-NK1.1 (clone PK-B6); PE-Cy7-conjugated anti-B220 (clone RA3-6B2); APC-conjugated anti-CD25 (clone PC61); biotin-conjugated anti-CD86 (clone GL1); biotin-conjugated anti-CD45 (clone 30-F11); anti-LAP (TGFβ1) (clone TW7-16B4); PE-conjugated anti-IFNγ (clone XMG1.2); PE-conjugated anti-IL4 (clone BVD6-24G2); PE-conjugated anti-IL10 (clone JES5-16E3); PE-conjugated anti-TNFα (clone MP6-XT22); PE-conjugated anti-IL6 (clone MP5-20F3); PE-conjugated anti-IL12p40 (clone C15.6); PE-conjugated anti-T-bet (clone 4B10); PE-conjugated anti-RORγt (clone Q31-378); PE-conjugated anti-GATA3 (clone L50-823); and FITC- or PE-conjugated anti-IgG1 (isotype control) (clone R3-34). The following mAbs from eBioscience (San Diego, CA, USA) were used: FITC- or APC-conjugated anti-CD19 (clone ID3); APC-conjugated anti-F4/80 (clone BM8); APC-conjugated anti-CD200R (clone OX110); PE-conjugated anti-IL17A (clone eBio17B7); PE-conjugated anti-iNOS (clone CXNFT); FITC- or PE-conjugated anti-Foxp3 (clone NRRF-30); and FITC- or PE-conjugated anti-FcεRI (clone MAR-1). The following mAbs from BioLegend were used: PE-conjugated anti-dectin-1 (clone RH1); FITC-conjugated anti-MR1 (clone C068C2). The following mAb from R&D Systems was used: PE-conjugated anti-arginase-1. The flow cytometric data were acquired using a FACSCalibur flow cytometer (Becton Dickson, San Jose, CA, USA) and were analyzed using FlowJo software (Tree Star, USA).

To perform surface staining, cells were harvested and washed twice with cold 0.5% BSA-containing PBS (FACS buffer). To block the Fc receptors, the cells were incubated with anti-CD16/CD32 mAbs on ice for 10 min and were subsequently stained with fluorescence-labeled mAbs. To perform intracellular staining, splenocytes were incubated with brefeldin A, an intracellular protein transport inhibitor (10 μg/ml), in RPMI medium for 2 hrs at 37°C. The cells were stained for cell surface markers, fixed with 4% PFA, washed once with cold FACS buffer, and permeabilized with 0.5% saponin. The permeabilized cells were then stained for an additional 30 min at room temperature with the indicated mAbs (PE-conjugated anti-IFNγ, anti-IL4, anti-IL10, anti-IL12p40, anti-IL6, anti-iNOS, anti-arginase-1; FITC-conjugated anti-IL17; or FITC or PE-conjugated isotype control rat IgG mAbs). Intracellular staining was performed according to the manufacturer’s protocol using the Foxp3 staining buffer set (eBioscience) with the indicated mAbs (PE-conjugated anti-LAP (TGFβ1), anti-T-bet, anti-GATA3, anti-RORγt, anti-TNFα or anti-Foxp3; FITC-conjugated anti-Foxp3; or FITC or PE-conjugated isotype control rat IgG mAbs). More than 5,000 cells per sample were acquired using FACSCalibur and analyzed with the FlowJo software package [24]. Gating strategies for flow cytometric analysis are following: Splenic and bone marrow-derived DCs were gated on CD11c^+^ populations; splenic and peritoneal macrophages were gated on CD11c^-^CD11b^+^F4/80^+^ populations; macrophages/microglia in the spinal cord were gated on CD45^+^CD11b^+^F4/80^+^ populations; Splenic mast cells were gated on FcεRI^+^CD200R^-^CD3^-^B220^-^ populations; Splenic basophils were gated on FcεRI^+^CD200R^+^CD3^-^B220^-^ populations; Splenic eosinophils were gated on Siglec-F^+^CD3^-^CD19^-^ populations; Splenic NKT cells were gated on CD3^+^NK1.1^+^ populations; Splenic CD4^+^ T cells were gated on CD3^+^CD4^+^ populations; CD4^+^ T cells in the spinal cord were gated on CD45^+^CD3^+^CD4^+^ populations; Treg cells in the spleen were gated on CD3^+^CD4^+^CD25^+^Foxp3^+^ populations; Treg cells in the spinal cord were gated on CD45^+^CD4^+^CD25^+^Foxp3^+^ population.

### Induction of EAE via active immunization

The encephalitogenic peptide MBP-Ac1-11 was used to induce EAE. The MBP-Ac1-11 peptide (Ac-ASQKRPSQRHG) was synthesized at 95% purity by Peptron (Daejeon, Korea). Active EAE was induced via subcutaneous injection with 150 μg of the MBP-Ac1-11 peptide in CFA containing 5 mg/ml of the heat-killed H37Ra strain of Mycobacterium tuberculosis (Difco Laboratories, Detroit, Michigan, USA) into the back and hind legs. Pertussis toxin (200 ng per mouse; Sigma, St. Louis, MO, USA) dissolved in PBS was administered intravenously on the day of and 48 hrs after immunization. The mice were scored for disease severity using the following EAE scoring scale: 0, no clinical signs; 1, limp tail; 2, paraparesis (weakness or incomplete paralysis of one or two hind limbs); 3, paraplegia (complete paralysis of both hind limbs); 4, paraplegia with fore limb weakness or paralysis; and 5, moribund state or death.

### Isolation of spinal cord mononuclear cells

Mice were anesthetized using ketamine and xylazine (40 mg/kg and 4 mg/kg, respectively) on day 21 or 24 after active immunization and were perfused through the left cardiac ventricle with cold phosphate-buffered saline (PBS, pH = 7.4) for 3 min to remove cells from the blood vessels. The spinal cord were removed, cut into small pieces using a scalpel and digested with 2.5 mg/ml collagenase type IV (Sigma, St. Louis, MO, USA) and 1 mg/ml DNase I (Promega, Madison, USA) for 15 min at 37°C. At the end of this incubation, the digested tissue was filtered through a 70-μm-pore cell strainer, and the cells were collected in a 50 ml Falcon tube and washed once with PBS containing 10% FBS (1400 rpm, 10 min, 4°C). The total cell suspension was then separated using a 37%/70% Percoll (GE Healthcare, Piscataway, NJ, USA) gradient, and mononuclear cells were collected from the layer between the 37% and 70% Percoll layers. After washing with PBS, the total mononuclear cell number was determined using a hemocytometer and 0.4% trypan blue (Welgene, Seoul, Korea) before staining.

### Isolation of peritoneal macrophages

Wild type and β-actin-SRG3 Tg B6 mice were i.p. injected with 2 ml of 4% thioglycollate broth (Sigma, St. Louis, MO, USA) in deionized water. Five days later, peritoneal macrophages were harvested from peritoneal lavage fluid and were subsequently plated on 24-well plates to select adherent macrophages. Two hrs later, the nonadherent cells were removed by washing with warm RPMI medium.

### Generation of bone marrow-derived DCs (BMDCs)

Bone marrow-derived dendritic cells (BMDCs) were generated from the bone marrow cells of mice as previously described [25]. Briefly, bone marrow cells from wild type (WT), CD2-SRG3 Tg, and β-actin-SRG3 Tg B6 mice were flushed with complete RPMI 1640 medium from femurs and tibiae of the indicated mice. After removal of red blood cells (RBCs) using ACK lysis buffer (0.15 M NH_4_Cl, 10 mM KHCO_3_, and 2 mM EDTA), the bone marrow cells were washed with PBS and seeded at a concentration of 1 x 10^6^ cells/ml in complete RPMI 1640 medium supplemented with recombinant mouse Flt3L (100 ng/ml; R&D Systems, Minneapolis,. MN, USA) in 24-well tissue culture plates. To generate BMDCs cultured with Flt3L, fresh cytokine-supplemented culture medium was added on day 5. On day 10, the BMDCs were harvested and subsequently stimulated with vehicle or LPS (40, 200, or 1000 ng/ml) for 14 hrs. The purity of CD11c^+^ cells was >93% after 10 days of culture.

### Quantitative reverse transcription-polymerase chain reaction (RT-PCR)

Total mRNAs were isolated from CD4^+^ cells using the Easy Red reagent (Intron, Korea) and were reverse-transcribed into cDNAs with oligo(dT) primers and M-MLV RT (Invitrogen Life Technologies, USA), according to the manufacturers’ instructions. PCR amplification was performed according to the recommended protocols for the PCR Amplification Kit; for each PCR amplification, 1 μg of cDNA, 5 pmol of each primer, and HiPi PCR Premix (ELPis Biotech, Korea) were included in a 20 μl reaction volume. PCR was conducted in an XP Thermal Cycler (Bioer Technology, Hangzhou, China) with the following thermal cycling parameters: 35 cycles of 94°C for 5 min, 94°C for 45 sec, 57°C for 45 sec, 72°C for 1 min, and 72°C for 5 min for SRG3 and 30 cycles of 94°C for 5 min, 94°C for 30 sec, 54°C for 30 sec, 72°C for 35 sec, and 72°C for 5 min for β-actin. Equal amounts of RT-PCR products were electrophoresed on a 1.5% agarose gel and stained with ethidium bromide. The intensity of the resulting bands was measured with a Gel Logic 100 Imaging System (Kodak, NY, USA) and analyzed using the Image J software package (National Institutes of Health, USA). The sequences of the PCR primers were as follow: β-actin forward, 5’-GTA TGG AAT CCT GTG GCA TC-3’ and β-actin reverse, 5’-AAG CAC TTG CGG TGC ACG AT-3’; SRG3 forward, 5’-GAC TAG ACC AAA CAT CTA CCT C-3’ and SRG3 reverse, 5’-GTC AAC TGA GCG ACT GGA TC-3’.

### Statistical analysis

Statistical significance was determined using Excel (Microsoft, USA). To compare two groups, Student’s t-test was performed. *P<0.05, **P<0.01, and ***P<0.001 were considered to be significant.

## Results

### β-actin promoter-driven over-expression of SRG3 in DCs suppresses LPS-induced pro-inflammatory cytokine production

**Fig 1:**
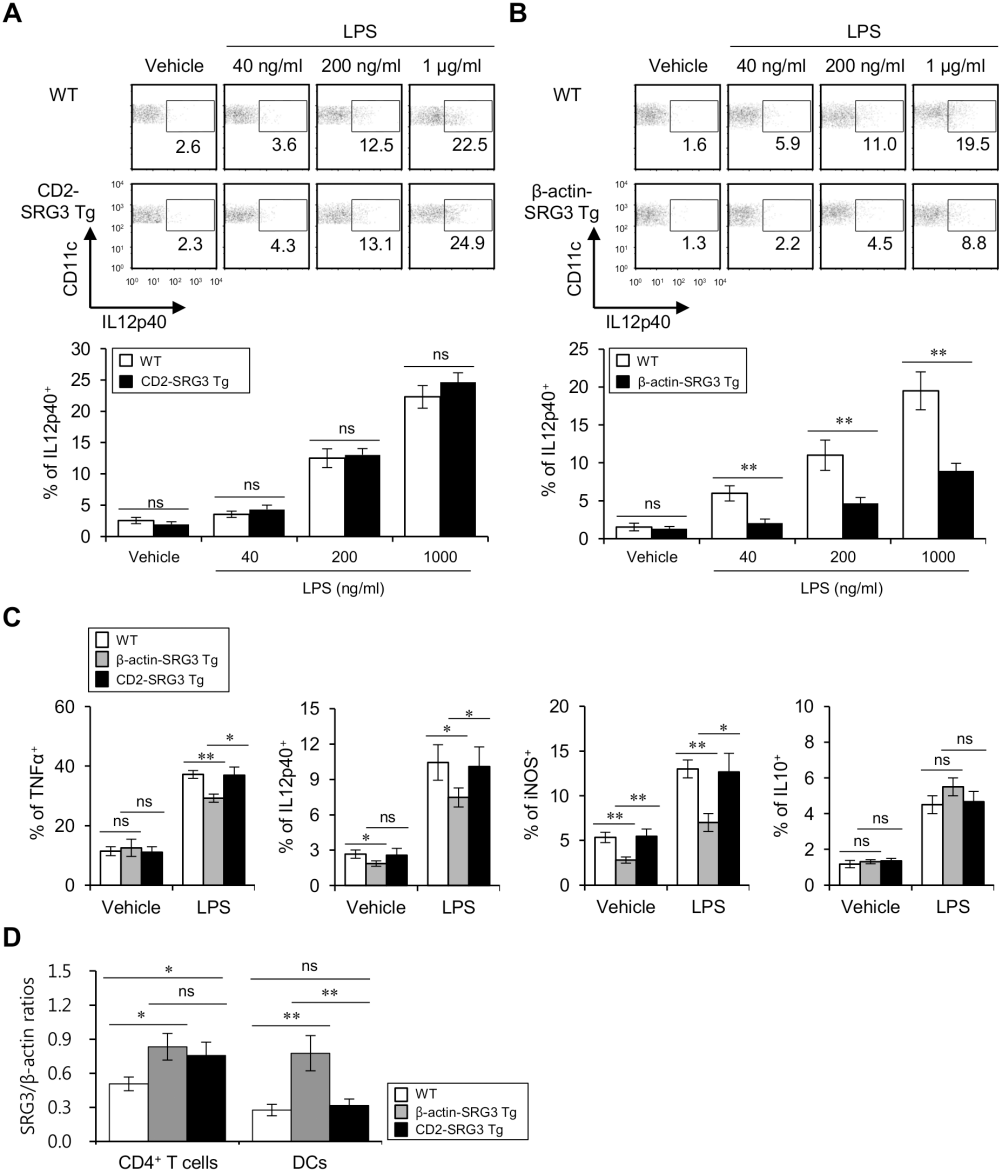
SRG3 over-expression driven by the β-actin promoter reduced cytokine production in DCs following LPS stimulation. Flt3L-cultured BMDCs (A) from both WT and CD2-SRG3 Tg B6 mice and (B) from both WT and β-actin-SRG3 Tg B6 mice were stimulated with either vehicle or LPS (40, 200, or 1000 ng/ml) for 14 hrs, and subsequently, the percentage of the IL12p40-expressing population among the CD11c^+^ BMDCs was assessed via flow cytometry. Representative data from three independent experiments are shown (upper panel). The graph in lower panel represents mean percentage ± SD for the proportion of IL12p40 (n = 3; **P<0.01). (C) WT, β-actin-SRG3 Tg, and CD2-SRG3 Tg mice were i.p. injected with LPS (2 μg) or vehicle. Sixteen hrs later, intracellular TNFα, IL12p40, iNOS, and IL10 production was assessed in splenic CD11c^+^ DCs via flow cytometry. The means ± SD are shown in the graphs (n = 4; *P<0.05, **P<0.01).

DCs play an important role in T cell polarization in the periphery and in the CNS. To determine whether the over-expression of SRG3 has any influence on DC activation elicited by LPS stimulation, BMDCs were generated from CD2-SRG3 Tg, β-actin-SRG3 Tg, and wild type (WT) B6 mice in the presence of Flt3L. After BMDCs were stimulated with LPS, their activation status was monitored based on their IL12p40 cytokine expression pattern. BMDCs from CD2-SRG3 Tg mice produced comparable levels of IL12p40 than those from WT B6 mice (Fig 1A). However, Intriguingly, BMDCs from β-actin-SRG3 Tg mice produced much lower levels of IL12p40 than those from WT B6 mice (Fig 1B), which suggested that the over-expression of SRG3 in DCs restrained the activation of DCs by reducing the production of pro-inflammatory cytokines such as IL12p40. Next, to determine whether SRG3 over-expression affects DC activation by LPS *in vivo*, β-actin-SRG3 Tg, CD2-SRG3 Tg, and WT B6 mice were i.p. injected with LPS (2 μg) or vehicle. Sixteen hrs later, the expression of TNFα, IL12p40, inducible nitric oxide synthase (iNOS), and IL10 in splenic DCs was examined via flow cytometric analysis. Strikingly, the expression levels of TNFα, IL12p40, and iNOS were decreased by SRG3 over-expression driven by the β-actin promoter. However, as expected, SRG3 over-expression driven by the CD2 promoter did not affect cytokine production in DCs because DCs from CD2-SRG3 Tg mice do not over-express SRG3, as these cells lack CD2 expression. In the vehicle-injected groups, DCs from β-actin-SRG3, but not CD2-SRG3 Tg mice expressed a much lower level of IL12p40 and iNOS but comparable levels of TNFα and IL10 compared to those from WT B6 mice, indicating that SRG3 over-expression in DCs maintains a low basal level of pro-inflammatory gene expression. In addition, no change in IL10 expression was observed between WT B6 mice and either CD2-SRG3 or β-actin-SRG3 Tg mice (Fig 1C; S1 Fig). As expected, the expression levels of SRG3 in splenic CD4^+^ T cells from both CD2-SRG3 Tg and β-actin-SRG3 Tg mice were up-regulated compared to those from WT B6 mice, whereas the expression levels of SRG3 in splenic DCs were up-regulated in β-actin-SRG3 Tg but not in CD2-SRG3 Tg mice compared to those from WT B6 mice (Fig 1D). These data demonstrated that SRG3 over-expression driven by the β-actin promoter, but not the CD2 promoter, led to the decreased expression of pro-inflammatory cytokines upon LPS stimulation both *in vivo* and *in vitro*.

### β-actin promoter-driven over-expression of SRG3 in macrophages enhances the differentiation of M2-type macrophages and inhibits M1 induction in response to LPS stimulation

**Fig 2:**
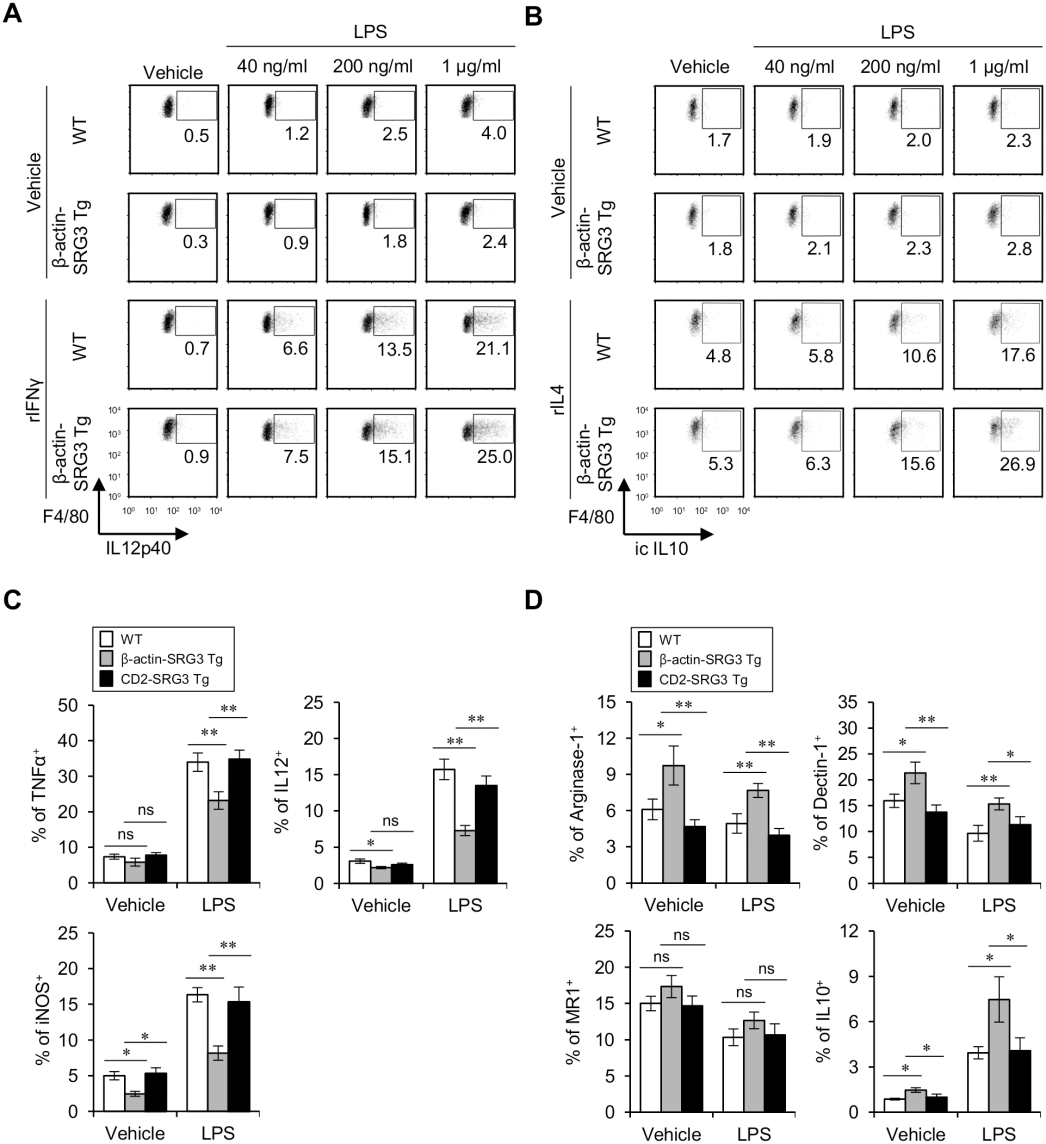
SRG3 over-expression driven by the β-actin promoter promotes the phenotypic shift of macrophages from M1 to M2 in response to LPS. (A-B) Peritoneal macrophages isolated from both WT and β-actin-SRG3 Tg B6 mice were primed for 5 hrs with either IFNγ (20 ng/ml) or IL4 (20 ng/ml). (A) IFNγ-primed macrophages were stimulated with either vehicle or LPS (40, 200, or 1000 ng/ml), and 12 hrs later, IL12p40 expression was analyzed via flow cytometry. Representative data from three independent experiments are shown. (B) IL4-primed macrophages were stimulated with either vehicle or LPS (40, 200, or 1000 ng/ml) for 12 hrs, and subsequently, intracellular IL10 production was analyzed via flow cytometry. Representative data are shown (n = 3). (C-D) WT, β-actin-SRG3 Tg and CD2-SRG3 Tg B6 mice were i.p. injected with LPS (2 μg) or vehicle. (C) Sixteen hrs later, intracellular TNFα, IL12p40, and iNOS production in splenic macrophages (CD11c^-^CD11b^+^F4/80^+^) was assessed via flow cytometry. The means ± SD are shown in the graphs (n = 4; *P<0.05, **P<0.01). (D) After 16 hrs of stimulation, intracellular arginase-1 and IL10 production and the surface expression of Dectin-1 and MR1 in macrophages (CD11c^-^CD11b^+^F4/80^+^) were assessed via flow cytometry. The means ± SD are shown in the graphs (n = 4; *P<0.05, **P<0.01).

Emerging evidence has demonstrated that macrophages are involved in many physiological regulatory functions, such as thermogenesis and wound healing, in addition to classical immune functions such as phagocytosis; the observation that macrophages perform multiple functions implies the existence of subsets of macrophages that perform distinct functions. In fact, macrophages are classified into two groups according to their cytokine profile; type 1 macrophages (M1) primarily produce IL12 and IL23, whereas type 2 macrophages (M2) produce IL10 and TGFβ [11]. Recently, it has been demonstrated that M2 macrophages modulate the development of autoimmune diseases such as EAE [26]. To investigate whether SRG3 expression in macrophages affects the phenotypes of these two distinct macrophage subsets, peritoneal macrophages from β-actin-SRG3 Tg and WT B6 mice were isolated and subsequently stimulated with IFNγ or IL4 *in vitro* to induce differentiation into M1 or M2 macrophages, respectively. We found that peritoneal macrophages from β-actin-SRG3 Tg mice produced IL12p40 at levels comparable to those of the control non-Tg mice. This result suggests that SRG3 over-expression does not affect the differentiation of IFNγ-stimulated peritoneal macrophages into the M1 phenotype (Fig 2A; Fig A in S2 Fig). However, we discovered that SRG3 expression significantly increased IL10-producing M2 macrophages, which might help CD4^+^ T cells to differentiate into Th2 types (Fig 2B; Fig B in S2 Fig). Whereas SRG3 over-expression in DCs down-regulated IL12p40 production, as shown in Fig 1A, SRG3 over-expression induced macrophages to differentiate into the IL10-producing M2 type. Taken together, these results strongly indicated that SRG3 over-expression in DCs or macrophages alters their capability to produce cytokines in a somewhat distinct manner. In addition, we examined whether the effect of SRG3 over-expression on M1/M2 polarization upon LPS-mediated activation was detectable *in vivo*. For this purpose, CD2-SRG3 Tg, β-actin-SRG3 Tg and WT B6 mice were i.p. injected with LPS (2 μg) or vehicle, and 16 hrs later, splenic macrophages were analyzed for the expression of M1 markers (TNFα, IL12p40, and iNOS) and M2 markers (arginase-1, Dectin-1, MR1, and IL10) via flow cytometry. Strikingly, SRG3 over-expression driven by the β-actin promoter suppressed the expression of M1 markers (TNFα, IL12p40, and iNOS) but enhanced the expression of M2 markers (arginase-1, Dectin-1, MR1, and IL10) in macrophages under both basal and LPS-activated conditions, although CD2 promoter-driven SRG3 over-expression did not affect the expression of M1 or M2 markers in macrophages. These results indicated that macrophages in CD2-SRG3 Tg mice do not overexpress SRG3 due to the lack of CD2 expression in macrophages (Fig 2C and 2D; Fig C in S2 Fig). Under LPS-stimulated conditions, the β-actin-SRG3 Tg mice displayed the down-regulation of most M2 markers (i.e., arginase-1, Dectin-1, and MR-1) except for IL10, whose production was increased in macrophages compared to the vehicle-treated conditions (Fig 2D; Fig C in S2 Fig). Thus, these results demonstrated that preferential polarization toward the M2 phenotype by β-actin promoter-driven SRG3 over-expression in macrophages was closely associated with the inhibition of M1-type gene expression, implying reciprocal regulation between the M1 and M2 subtypes.

### β-actin promoter-driven, but not CD2 promoter-driven, SRG3 over-expression increases the number of basophils and mast cells and the production of IL4

**Fig 3:**
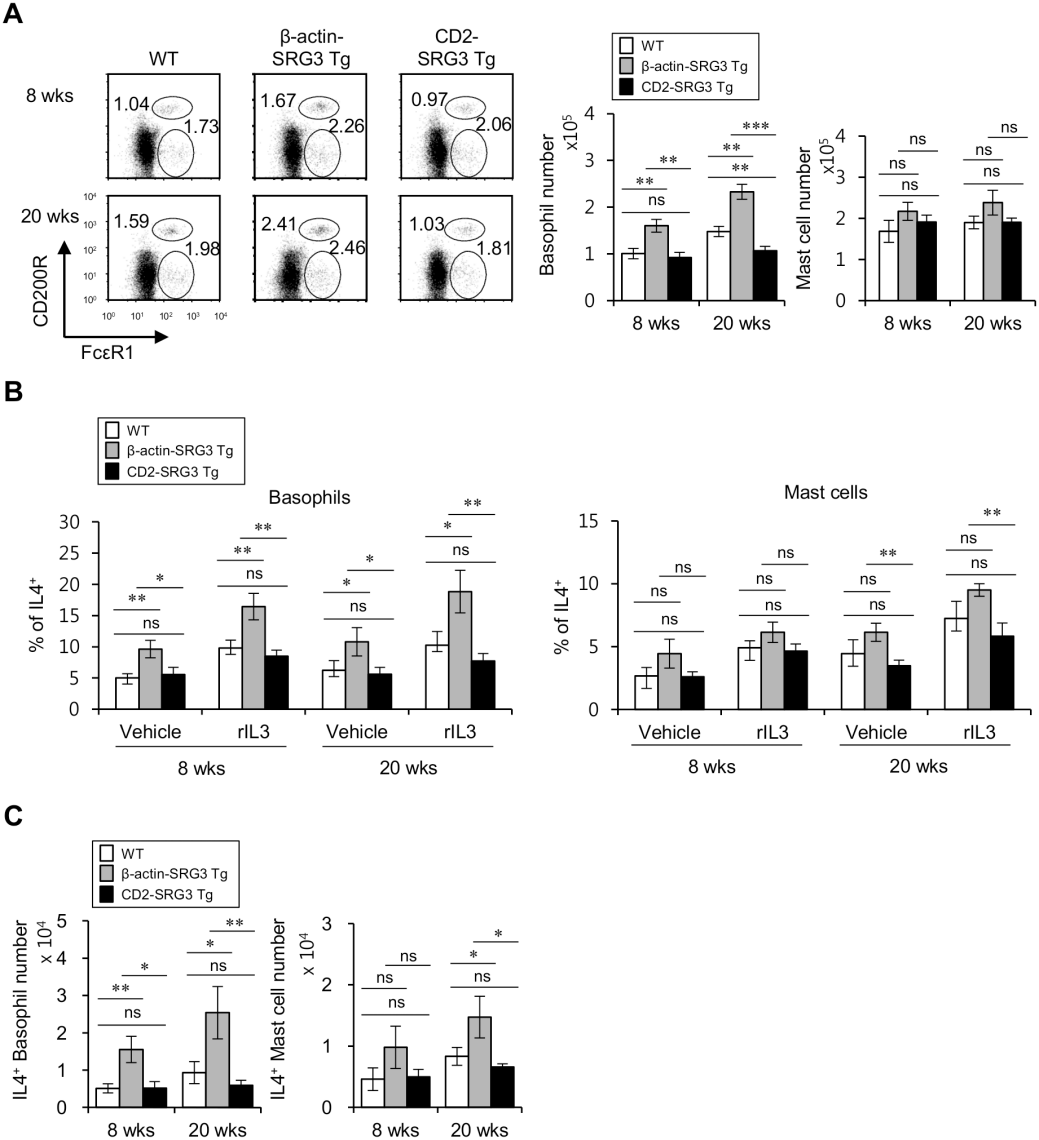
β-actin promoter-driven SRG3 over-expression increases both the basophil and mast cell numbers and IL4 production. (A) Splenocytes were isolated from the spleens of WT, CD2-SRG3 Tg, and β-actin-SRG3 Tg B6 mice at either 8 or 20 weeks of age. The frequencies of both mast cells (FcεRI^+^CD200R^-^CD3^-^B220^-^) and basophils (FcεRI^+^CD200R^+^CD3^-^B220^-^) in the spleen were plotted (upper left panel). The absolute cell numbers of both basophils and mast cells were determined (upper right panels). The means ± SD are shown (n = 3–4; **P<0.01, ***P<0.001). (B) Splenocytes from WT, CD2-SRG3 Tg, and β-actin-SRG3 Tg B6 mice at the age of either 8 or 20 weeks were cultured in the presence of recombinant mIL3 (20 ng/ml) for 24 hrs. The percentages of IL4-producing cells among both basophils and mast cells were analyzed via flow cytometry. The mean values ± SD are shown (n = 3–4; *P<0.05, **P<0.01). (c) Splenocytes were isolated from the spleens of WT, CD2-SRG3 Tg, and β-actin-SRG3 Tg B6 mice at either 8 or 20 weeks of age. The absolute cell numbers of both IL4^+^ basophils and IL4^+^ mast cells were determined. The means ± SD are shown (n = 3–4; **P<0.01, ***P<0.001).

IL4 is a cytokine that induces the M2 polarization of macrophages [11], and basophil-derived IL4 has been reported to represent an effector of the generation of M2 macrophages [27]. Because basophils and mast cells are capable of producing IL4, we examined whether the over-expression of SRG3 modulates the frequencies of basophils and mast cells and the levels of IL4 secretion by these cells in the absence or presence of rIL3 stimulation. Based on the previous study [28], splenic basophils were gated on FcεRI^+^CD200R^+^CD3^-^B220^-^ populations mostly expressing basophil marker DX5 and could be distinguished from splenic mast cells gated on FcεRI^+^CD200R^-^CD3^-^B220^-^ populations expressing mast cell marker c-kit (S3 Fig). We found that even under unstimulated conditions, the over-expression of SRG3 driven by the β-actin promoter, but not the CD2 promoter, elevated the number of basophils and mast cells in the spleen. Moreover, 20-week-old β-actin SRG3 Tg mice displayed a significantly higher number of basophils than 8-week-old mice, whereas the relative increase in the number of basophils from 8 to 20 weeks of age was similar between the β-actin-SRG3 Tg and WT B6 mice. In contrast, the CD2-SRG3 Tg mice exhibited the suppressed development of basophils compared with WT B6 mice at 20, but not 8, weeks of age (Fig 3A). Furthermore, splenic basophils from β-actin-SRG3, but not CD2-SRG3, Tg mice produced a larger amount of IL4 in both the absence and presence of rIL3 stimulation than those from WT B6 mice, and similarly, mast cells showed a tendency towards increased IL4 production under both conditions (Fig 3B). Under unstimulated conditions, the over-expression of SRG3 driven by the β-actin promoter, but not the CD2 promoter, elevated the number of IL4-producing basophils in the spleen at 8 weeks of age, whereas β-actin-SRG3 Tg mice but not CD2-SRG3 Tg mice displayed a significantly higher number of both IL4-producing basophils and IL4-producing mast cells than WT B6 mice at 20-week-old age (Fig 3C). However, both β-actin-SRG3 and CD2-SRG3 Tg mice showed no significant difference in cell number and IL4 production of eosinophils and NKT cells (S4 Fig). These results indicate that the expansion and increased IL4 production of basophils and mast cells were correlated with anti-inflammatory function by SRG3 over-expression.

### Differential outcome of EAE pathogenesis depending on the SRG3 over-expression pattern in MBP-specific TCR transgenic B10.PL mice: the development of EAE was restrained in the β-actin-SRG3 Tg mice but was facilitated in the CD2-SRG3 Tg mice

**Fig 4:**
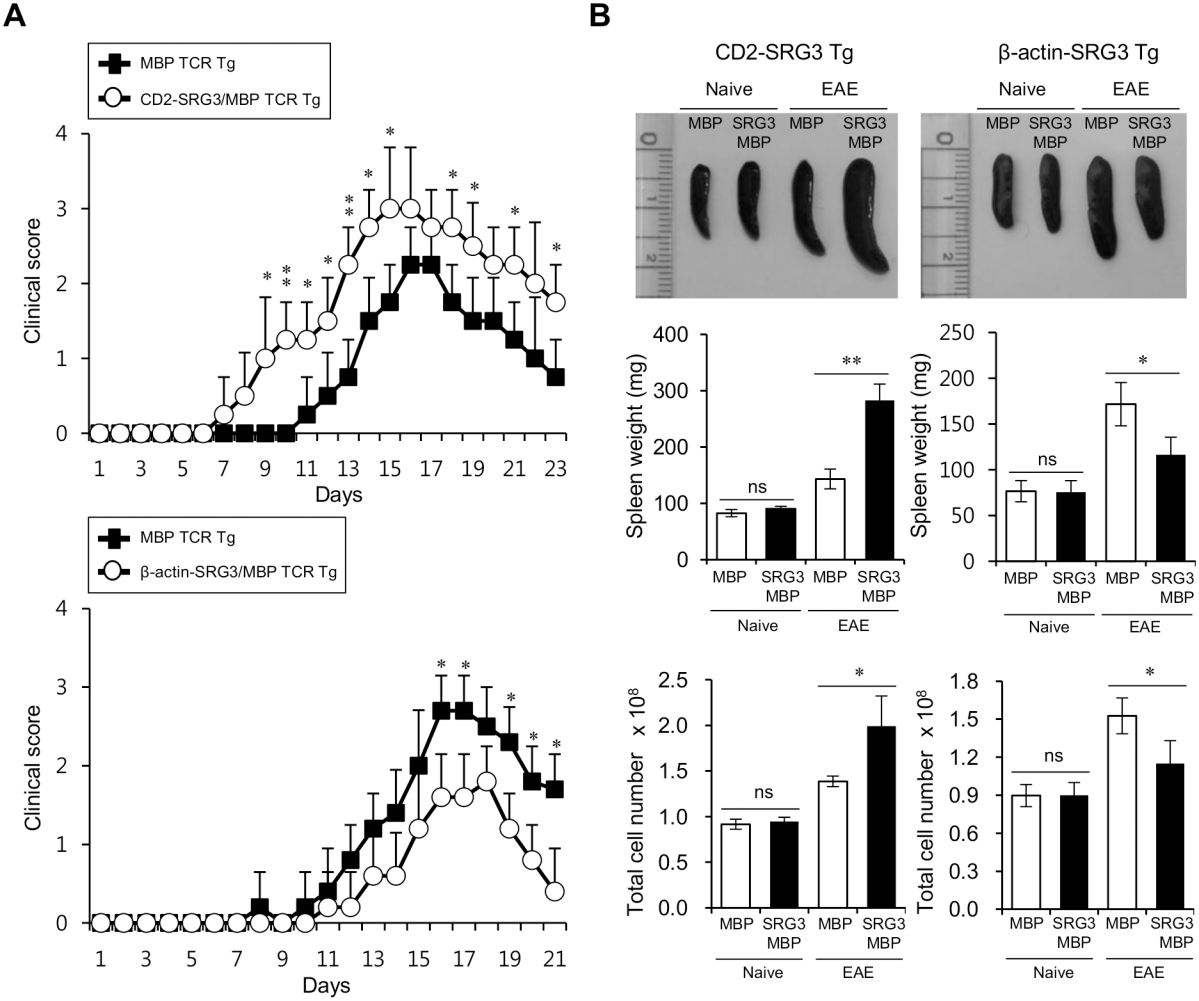
Differential outcome of EAE pathogenesis depending on the SRG3 over-expression pattern in MBP-specific TCR transgenic B10.PL mice: the development of EAE was suppressed in β-actin-SRG3 Tg mice but was facilitated in CD2-SRG3 Tg mice. (A-B) Both MBP TCR Tg B10.PL mice and CD2-SRG3/MBP TCR double Tg B10.PL mice (upper panel) or both MBP TCR Tg B10.PL mice and β-acin-SRG3/MBP TCR double Tg B10.PL mice (lower panel) were either non-immunized or s.c. immunized with the MBP-Ac1-11 peptide in CFA. (A) Subsequently, the disease severity was monitored daily according to the classical EAE scoring system after immunization; one representative result out of two independent experiments is shown as the mean ± SD of 5 mice per group (*P<0.05, **P<0.01). (B) Spleens were prepared from the aforementioned four experimental groups, and corresponding photographs were captured (upper panel). Additionally, spleen weight (the middle panel) and total cell number (the lower panel) are shown as the mean values ± SD (n = 5; *P<0.05, **P<0.01).

A previous study showed that SRG3 over-expression driven by the CD2 promoter facilitates MOG peptide-induced EAE development in B6 mice [7]. To confirm whether SRG3 over-expression accelerates the outcome of EAE in self-antigen-specific TCR Tg mice, which carry a more EAE-susceptible genetic background, we subjected MBP TCR transgenic (Tg) B10.PL mice (u haplotype) to MBP peptide-induced EAE. First, we examined whether CD2-SRG3 over-expression exacerbated EAE in MBP TCR Tg mice. As expected, CD2-SRG3/MBP TCR double Tg B10.PL mice developed EAE more severely and rapidly than MBP TCR single Tg mice (Fig 4A, upper panel). Because β-actin-SRG3 Tg mice are in the B6 (H2^b^) genetic background, next, these mice were backcrossed more than 7 times with MBP TCR Tg B10.PL (H2^u^) mice to generate β-actin-SRG3/MBP TCR double Tg mice carrying the H2^u^ haplotype, which is well known to increase the susceptibility to EAE induction via MBP antigen immunization. Using these double Tg mice, we investigated whether distinct modes of SRG3 over-expression significantly influence the outcome of EAE upon MBP peptide immunization. Surprisingly, we found that the ubiquitous over-expression of SRG3 in β-actin-SRG3 Tg mice attenuated the severity and rate of EAE development compared to the endogenous expression of SRG3 in MBP TCR single Tg control mice (Fig 4A, lower panel).

Furthermore, upon MBP peptide immunization, CD2-SRG3/MBP TCR double Tg mice showed a significantly increased spleen weight and displayed an increased number of splenocytes compared with the control mice, indicating that T cells from these mice were hyper-activated to proliferate (Fig 4B, left panel). Alternatively, the β-actin-SRG3 over-expressing mice did not show any dramatic increase in either spleen weight or the number of splenocytes compared with the control mice (Fig 4B, right panel). However, under non-immunization conditions, the CD2-SRG3 and β-actin-SRG3-overexpressing mice showed no significant difference in spleen weight or the number of splenocytes compared with the MBP TCR single Tg control mice (Fig 4B). These results strongly indicated that distinct mode of SRG3 over-expression affect the outcome of EAE in mice.

### During EAE development, SRG3 over-expression driven by the CD2 promoter promotes Th1 and Th17 differentiation, whereas SRG3 over-expression driven by the β-actin promoter increases Th2 differentiation but decreases Th1 and Th17 differentiation

**Fig 5:**
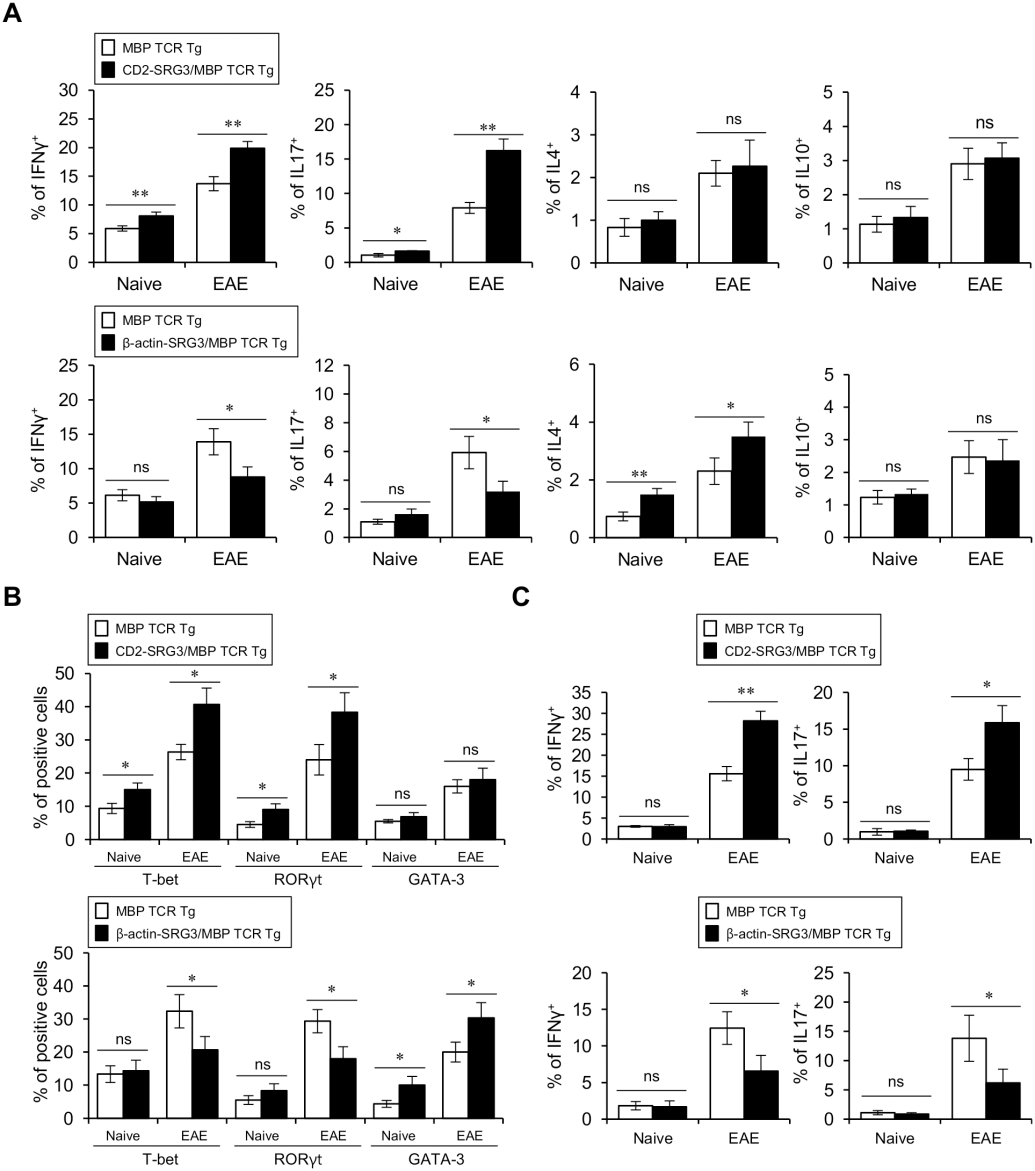
During EAE development, SRG3 over-expression driven by the CD2 promoter enhances Th1 and Th17 differentiation, whereas SRG3 over-expression driven by the β-actin promoter increases Th2 differentiation but decreases Th1 and Th17 differentiation. (A-C) Both MBP TCR Tg B10.PL mice and CD2-SRG3/MBP TCR double Tg B10.PL mice (upper panel in Fig 5A, 5B, and 5C) or both MBP TCR Tg B10.PL mice and β-actin-SRG3/MBP TCR double Tg B10.PL mice (lower panel in Fig 5A, 5B, and 5C) were either non-immunized or s.c. immunized with the MBP-Ac1-11 peptide in CFA. (A) Purified CD4^+^ splenocytes from the four groups were activated using plate-bound anti-CD3 (10 μg/ml) and anti-CD28 (1 μg/ml) mAbs for 16 hrs and subsequently stimulated with PMA/ionomycin for 2 hrs in the presence of brefeldin A (10 μg/ml). The intracellular expression of IFNγ, IL17, IL4, and IL10 was analyzed via flow cytometry. The mean values ± SD are shown (n = 5; *P<0.05, **P<0.01). (B) The expression of T-bet, RORγt, and GATA-3 in splenic CD4^+^ T cells purified from the four groups was analyzed via flow cytometry. The mean values ± SD are shown (n = 5; *P<0.05). (C) Mononuclear cells (MNCs) were isolated from the spinal cord of the four groups using a Percoll gradient on day 21 or 24 after EAE induction. The isolated MNCs were incubated for 2 hrs in brefeldin A (10 μg/ml), and subsequently, the intracellular expression of the cytokines IFNγ and IL17 was analyzed via flow cytometry. The mean values ± SD are shown (n = 5; *P<0.05, **P<0.01).

Because CD2-SRG3 over-expression facilitates the development of EAE, we examined whether CD2-SRG3 over-expression polarizes CD4^+^ T cells toward the Th1 and Th17 phenotypes since it has been accepted that EAE pathogenesis is primarily mediated by both Th1 and Th17 immune responses [29]. Thus, we measured the cytokine expression pattern of CD4^+^ T cells in both the spleen and the spinal cord under either naive (no MBP immunization) or EAE conditions (MBP immunization) in CD2-SRG3/MBP TCR double Tg mice. We found that the expression of both IFNγ and IL17 was significantly increased but that the expression of both IL4 and IL10 was comparable in splenic CD4^+^ T cells from CD2-SRG3-over-expressing mice compared to those from MBP TCR single Tg B10.PL mice (Fig 5A, upper panel; Fig A in S5 Fig). Next, we hypothesized that β-actin promoter-driven SRG3 over-expression influences CD4^+^ T cell differentiation during EAE development due to differential effects of the SRG3 expression pattern on EAE pathogenesis. To test this hypothesis, we examined the cytokine expression pattern of T helper cells after immunization. We found that the frequency of Th1 and Th17 cells was dramatically decreased in β-actin-SRG3/MBP TCR double Tg mice compared to the control mice and that IL4-producing Th2 cells were increased in both the naive (non-immunization) and EAE (immunization) groups in these double Tg mice compared to the control mice (Fig 5A, lower panel; Fig B in S5 Fig). Based on these cytokine profiles, we evaluated the Th1/Th2 ratio among splenic CD4^+^ T cells and found that it significantly increased in CD2-SRG3/MBP TCR double Tg mice but dramatically decreased in β-actin-SRG3/MBP TCR double Tg mice compared to the control mice (Fig A in S6 Fig). In addition, we compared the intracellular expression of specific transcription factors that orchestrate the differentiation of T helper subsets in CD4^+^ T cells between the CD2-SRG3 or β-actin-SRG3 Tg mice and the WT mice. We examined T-bet for Th1 cells, GATA-3 for Th2 cells, and RORγt for Th17 cells [30]. The CD2-SRG3 Tg mice showed increased numbers of CD4^+^ T cells expressing T-bet and RORγt but not GATA-3 (Fig 5B, upper panel). As expected, the ubiquitous expression of SRG3 down-regulated T-bet and RORγt expression but up-regulated GATA-3 expression; these results were consistent with the cytokine profiles of T helper cells (Fig 5B, lower panel). Finally, we observed a dramatic increase in the frequencies of Th1 and Th17 cells that infiltrated into the spinal cord in the CD2-SRG3/MBP TCR double Tg mice subjected to EAE compared to the control mice (Fig 5C, upper panel). Next, we examined whether β-actin promoter-mediated SRG3 over-expression restricts the infiltration of pathogenic effector T cells in the spinal cord during EAE development. We found that the infiltration of Th1 and Th17 cells into the spinal cord was significantly reduced in the β-actin-SRG3/MBP TCR double Tg mice subjected to EAE compared to the control mice (Fig 5C, lower panel). Taken together, our results demonstrated that CD2-SRG3-overexpressing mice are much more vulnerable to the pathogenesis of autoimmune diseases such as EAE but that the over-expression of SRG3 in additional cell types inhibits EAE development.

### The contrasting effects of differential SRG3 over-expression on the severity of EAE were associated with the alteration of the phenotypes of dendritic cells and macrophages

**Fig 6:**
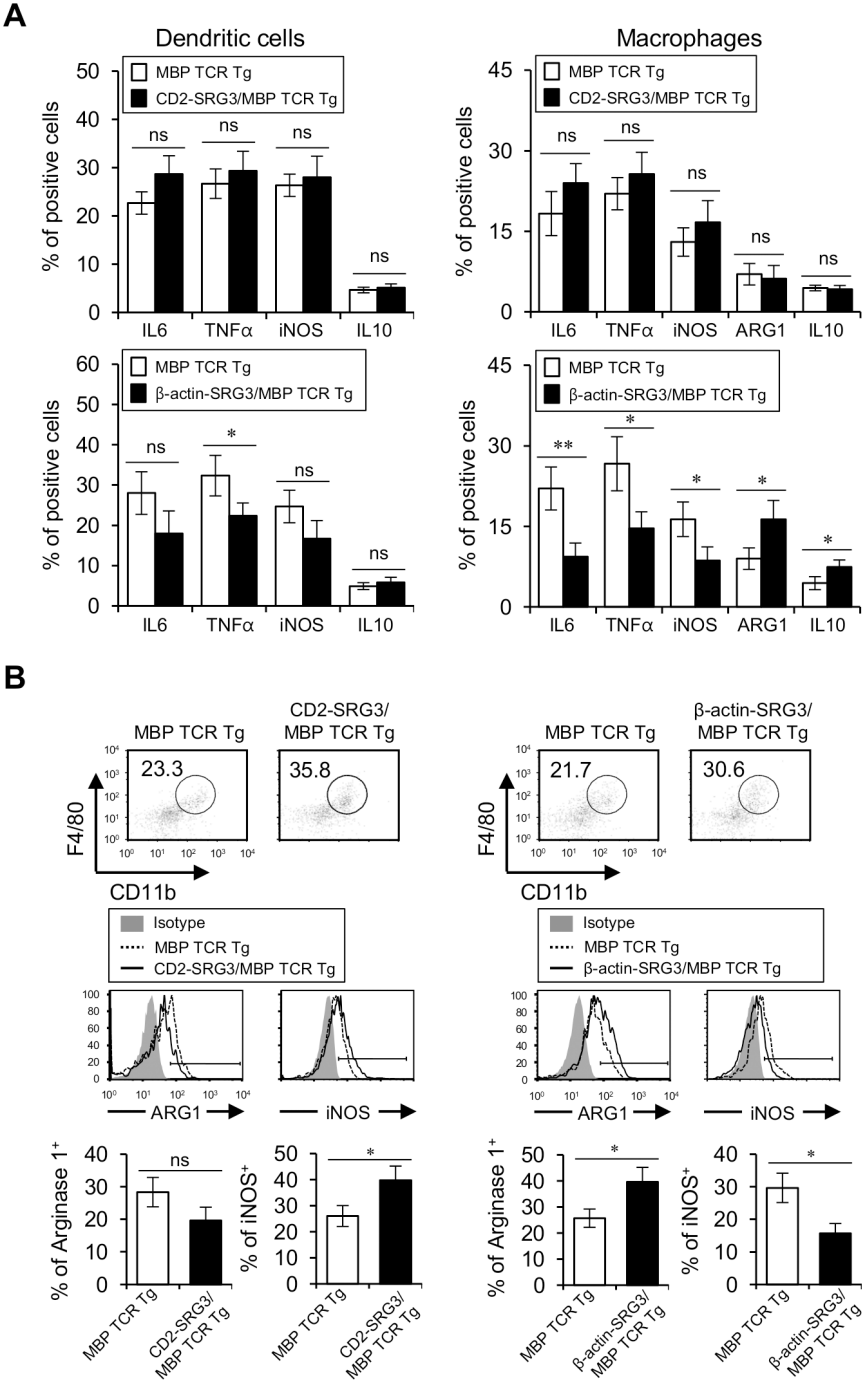
The contrasting effects of differential SRG3 over-expression on the severity of EAE were associated with the alteration of the phenotypes of DCs and macrophages. (A) Splenocytes were prepared from both MBP TCR Tg B10.PL mice and CD2-SRG3/MBP TCR double Tg B10.PL mice immunized with MBP to induce EAE. The intracellular production of IL6, TNFα, iNOS, and IL10 was assessed in splenic CD11c^+^ DCs via flow cytometry (upper left panel). Additionally, intracellular IL6, TNFα, iNOS, arginase-1, and IL10 production was assessed in splenic macrophages (CD11c^-^CD11b^+^F4/80^+^) via flow cytometry (upper right panel). Alternatively, splenocytes were prepared from both MBP TCR Tg B10.PL mice and β-actin-SRG3/MBP TCR double Tg B10.PL mice immunized with MBP to induce EAE. Intracellular IL6, TNFα, iNOS, and IL10 production was assessed in splenic DCs (CD11c^+^) (lower left panel) and intracellular IL6, TNFα, iNOS, arginase-1, and IL10 production was assessed in splenic macrophages (CD11c^-^CD11b^+^F4/80^+^) via flow cytometry (lower right panel). The mean values ± SD are shown (n = 5; *P<0.05, **P<0.01). (B) Mononuclear cells (MNCs) were isolated from the spinal cord of both MBP TCR Tg B10.PL mice and CD2-SRG3/MBP TCR double Tg B10.PL mice immunized with MBP to induce EAE. The intracellular expression of arginase-1 and iNOS was assessed in macrophages/microglia (CD45^+^CD11b^+^F4/80^+^) via flow cytometry (left panel). Alternatively, MNCs were isolated from the spinal cord of both MBP TCR Tg B10.PL mice and β-actin-SRG3/MBP TCR double Tg B10.PL mice immunized with MBP to induce EAE. The intracellular expression of arginase-1 and iNOS was assessed in macrophages/microglia (CD45^+^CD11b^+^F4/80^+^) via flow cytometry (right panel). The mean values ± SD are shown (n = 5; *P<0.05).

Previously, it has been demonstrated that APCs such as DCs and macrophages play important roles in the pathogenesis of EAE. Specifically, the response and polarization of macrophages and microglia in the CNS are directly involved in the pathogenesis of EAE [12]. Clearly, DCs also participate in the pathological process by presenting self-antigens to CD4^+^ T cells during EAE. Therefore, we investigated the impact of SRG3 over-expression on DC and macrophage activation during EAE induction. During EAE pathogenesis, CD2 promoter-driven SRG3 over-expression slightly increased the expression of IL6, TNFα, and iNOS, whereas β-actin promoter-driven SRG3 over-expression attenuated the expression of IL6, TNFα, and iNOS. In addition, in the macrophage population, the effect of SRG3 over-expression on macrophage differentiation was remarkable. Regarding the expression of M1 markers (IL6, TNFα, and iNOS) and M2 markers (arginase-1 and IL10), CD2 promoter-driven SRG3 over-expression slightly increased the expression of M1 markers but not M2 markers, whereas β-actin promoter-driven SRG3 over-expression significantly decreased the expression of M1 markers and dramatically increased the expression of M2 markers (Fig 6A). It has been reported that a shift towards the pro-inflammatory M1 phenotype promotes severe EAE but that a shift towards the M2 phenotype suppresses EAE in macrophages and microglia [26, 31]. Thus, we examined whether the effect of SRG3 over-expression on the macrophage differentiation affects macrophages in the spinal cord of EAE-induced mice. As expected, CD2 promoter-driven SRG3 over-expression in EAE-induced mice promoted the M1 phenotype (iNOS) and suppressed the M2 phenotype (arginase-1) in macrophages that infiltrated the spinal cord compared to endogenous SRG3 expression. In contrast, β-actin promoter-driven SRG3 over-expression shifted macrophages from the classical M1 type toward the M2 phenotype in the spinal cord of EAE-induced mice (Fig 6B). Although the numbers of both M1 and M2 macrophages infiltrated into the spinal cord were significantly increased in CD2-SRG3/MBP TCR double Tg mice compared to the control mice, proportion of M1 macrophages was up to two times higher than M2 macrophages. However, the number of M1 but not M2 macrophages infiltrated into the spinal cord was significantly reduced in β-actin-SRG3/MBP TCR double Tg mice compared to the control mice (Fig A in S7 Fig). It has been recently shown that an imbalance of macrophage M1–M2 polarization is often associated with various diseases or inflammatory conditions [32]. M1/M2 ratio among macrophages/microglia from the spinal cord but not the spleen was significantly increased in CD2-SRG3/MBP TCR double Tg mice compared to the control mice, but M1/M2 ratio among those from both the spleen and spinal cord decreased in β-actin-SRG3/MBP TCR double Tg mice compared to the control mice (Fig B in S7 Fig). Thus, our results showed that the contrasting effects between CD2 and β-actin promoter-driven SRG3 over-expression on the severity of EAE were associated with the alteration of the M1/M2 ratio and the inflammatory phenotype of DCs.

### Infiltration of Treg cells into the spinal cord was dramatically increased in β-actin-SRG3 Tg mice compared to CD2-SRG3 Tg mice during EAE pathogenesis

**Fig 7:**
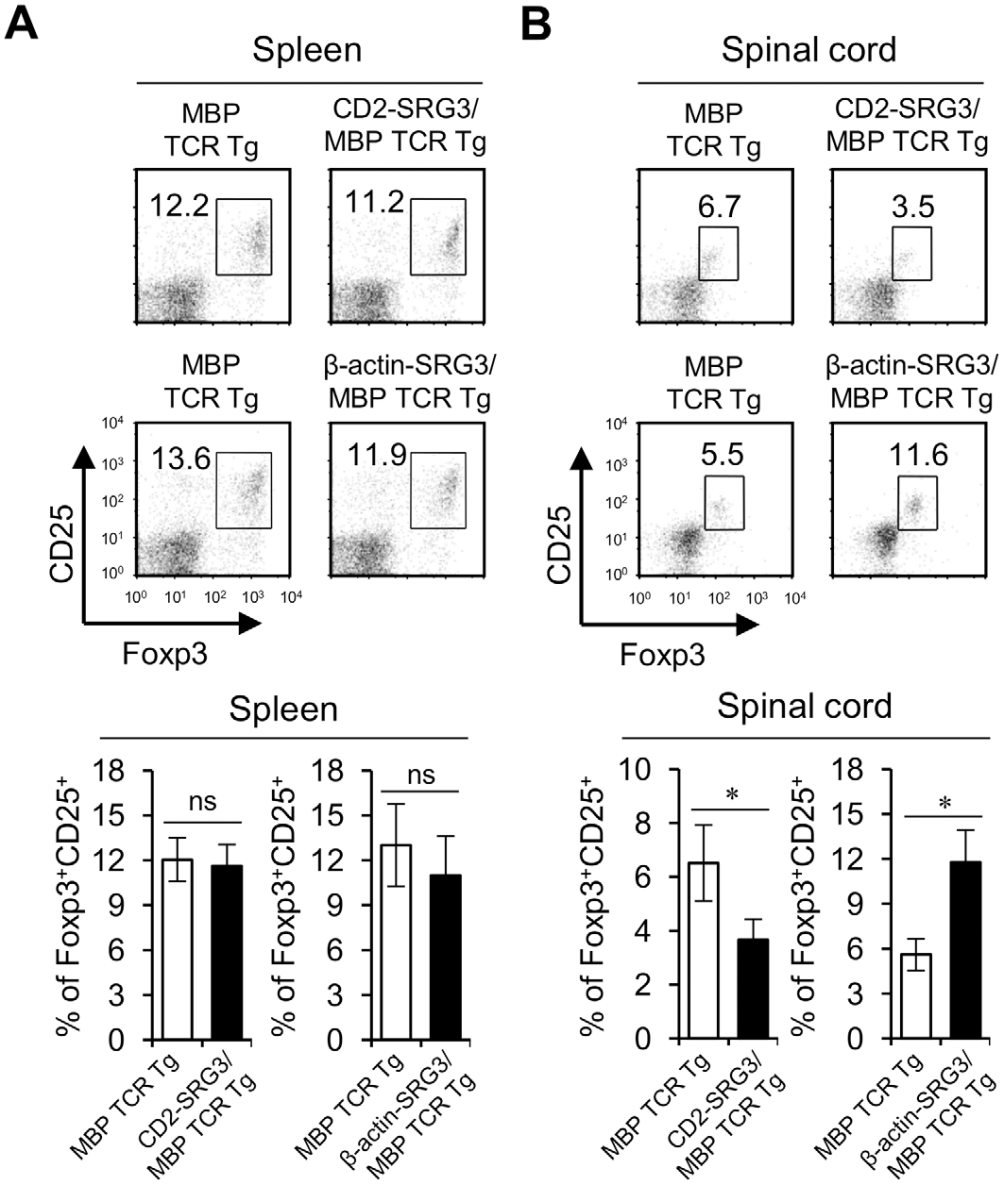
Infiltration of Treg cells into the spinal cord was dramatically increased in β-actin-SRG3 Tg mice compared to CD2-SRG3 Tg mice during EAE pathogenesis. (A) Splenocytes and (B) MNCs were prepared from the spinal cord of both MBP TCR Tg B10.PL mice and CD2-SRG3/MBP TCR double Tg B10.PL mice immunized with MBP to induce EAE. The percentages of CD25^+^FoxP3^+^ Treg cells among the total CD4^+^ T cell population in splenocytes and MNCs were evaluated via flow cytometry (upper panel). Alternatively, splenocytes and MNCs were prepared from the spinal cord of both MBP TCR Tg B10.PL mice and β-actin-SRG3/MBP TCR double Tg B10.PL mice immunized with MBP to induce EAE. The percentages of CD25^+^FoxP3^+^ Treg cells among the total CD4^+^ T cell population in splenocytes and MNCs were evaluated via flow cytometry (lower panel). The mean values ± SD are shown (n = 5; *P<0.05).

Recently, it has been reported that Treg cells play a vital role in maintaining the homeostasis of the immune system during EAE development [20, 21]. Thus, we examined whether the discrepant outcome between CD2-SRG3 and β-actin-SRG3 Tg mice is attributable to a difference in the Treg cell frequencies among the CD4^+^ T cells in the spleen and the spinal cord. We found that no significant difference in the splenic Treg cell populations between the CD2-SRG3 and β-actin-SRG3 Tg mice. However, interestingly, after EAE induction, the frequency of spinal cord-infiltrating Treg cells was significantly increased in the β-actin-SRG3/MBP TCR double Tg mice but was dramatically reduced in the CD2-SRG3/MBP TCR double Tg mice compared to the control mice (Fig 7). Recently it has been reported that the imbalance between Th17 and Treg cells critically contributes to development of autoimmune diseases such as arthritis and EAE [33]. To evaluate if this applies to our findings, we measured the frequency of Th17 cells in the spleen and spinal cord and observed that Th17/Treg ratio among CD4^+^ T cells from both the spleen and spinal cord was significantly increased in CD2-SRG3/MBP TCR double Tg mice but decreased in β-actin-SRG3/MBP TCR double Tg mice compared to MBP TCR Tg control mice (Fig B in S6 Fig). Thus, the contrasting effect of differential SRG3 over-expression on the Treg cell frequency in the spinal cord shows a strong association between the Treg cell phenotype and the severity of EAE pathogenesis.

## Discussion

Although one study has recently reported that the over-expression of SRG3 driven by the CD2 promoter exacerbated EAE upon immunization with MOG self-antigens [7], the cellular mechanisms by which ubiquitous SRG3 up-regulation in multiple cell types and by which CD2 promoter-restricted SRG3 over-expression in T cells differentially influence EAE development remain largely unknown. Therefore, we aimed to determine the contribution of the ubiquitous over-expression of SRG3 driven by the β-actin promoter to EAE induction. To investigate the effects of the differential expression of SRG3 on EAE pathogenesis, we crossed CD2-SRG3 and β-actin-SRG3 Tg mice with MBP TCR Tg mice carrying the H2^u^ haplotype in the B10.PL genetic background, which is much more susceptible to EAE induction, to generate CD2-SRG3 and β-actin-SRG3 MBP TCR double Tg mice, respectively.

Employing these newly generated double Tg mice, herein, we have demonstrated that the over-expression of SRG3 driven by the CD2 promoter accelerated the development of EAE upon immunization with MBP self-antigens. The proportions of both the IL17- and IFNγ-producing populations among CD4^+^ T cells isolated from the spleen or the spinal cord of CD2-SRG3/MBP TCR double Tg mice were much higher than those of control mice following EAE induction. Consistent with a previous report that the infiltration of Th1 and Th17 cells resulted in demyelination in the CNS during the progression of EAE [29], the facilitated development of EAE in the CD2-SRG3/MBP TCR double Tg mice was supported by an increased number of CNS-infiltrating Th1 and Th17 cells.

In the experiments using β-actin-SRG3/MBP TCR double Tg mice, we unexpectedly found that the ubiquitous expression of SRG3 inhibited the development of EAE. Based on our data, the over-expression of SRG3 driven by the β-actin promoter down-regulates Th1/Th17 differentiation but up-regulates Th2 differentiation. The contrasting effects between CD2 and β-actin promoter-driven SRG3 over-expression on EAE development might be associated with the alteration of the pro-inflammatory activation of DCs or M1/M2 phenotypes of macrophages because these innate immune cells modulate adaptive immune responses.

As APCs, DCs and macrophages are considered to be able to activate auto-reactive T cells under inflammatory conditions such as EAE [9]. Because DCs and macrophages from the spleen of CD2-SRG3 Tg mice display a tendency toward increased production of inflammatory cytokines, these cells might contribute to the induction of Th1/Th17 differentiation upon MBP peptide/CFA immunization. This hypothesis was further supported by an *in vitro* LPS stimulation experiment using splenic and BMDCs from CD2-SRG3 Tg mice. However, CD2 promoter-driven SRG3 over-expression shifted M2 macrophage/microglial differentiation toward the M1 phenotype in the spinal cord. These results indicated that the CD4^+^ T cell-dependent shift of the differentiation of macrophages/microglia from M2 to M1 contributes to accelerated CNS inflammation due to CD2 promoter-driven SRG3 over-expression in EAE.

In contrast, β-actin promoter-driven SRG3 over-expression in BMDCs and splenic DCs inhibited pro-inflammatory cytokine production in response to LPS. These data support the concept that during EAE pathogenesis, β-actin promoter-driven SRG3 over-expression might contribute to the restriction of CD4^+^ T cells from differentiating into pathogenic Th1 and Th17 cells via the regulation of DC function. In addition, compared to classical M1 macrophages, M2 macrophages, which exhibit poor antigen-presenting capacities, have been demonstrated to contribute to remyelination and tissue repair in the CNS during the spontaneous recovery phase of EAE progression [12, 34]. Particularly, the phenotypic switch of macrophages from M1 to M2 in the spleen and the spinal cord due to β-actin promoter-driven SRG3 over-expression may contribute to the accelerated recovery from EAE symptoms based on our data. In fact, H&E-stained paraffin sections of the spinal cords from EAE-induced β-actin-SRG3/MBP TCR double Tg mice (H2^b/u^ haplotype) displayed a decrease in immune cell infiltration compared with those from EAE-induced control mice (data not shown), implying the potential function of SRG3 over-expression in the repair of damaged CNS tissue in EAE-induced mice.

However, the mechanism by which macrophages in the CNS (microglia) differentiate into the anti-inflammatory M2 phenotype remains unclear. It has been reported that the alternative activation of microglial cells by IL4, primarily driven by CNS-resident microglia, safely protects against inflammation and suppresses the number of infiltrating inflammatory cells [17]. Based on our results, we speculate that IL4 produced by basophils and mast cells in β-actin-SRG3 Tg mice might facilitate the differentiation of macrophages (i.e., peritoneal macrophages) into the M2 phenotype, leading to greater IL10 production in these mice than in WT mice. This SRG3 over-expression-induced alteration in the cytokine production pattern may contribute to an enhanced shift of immune responses to the Th2 phenotype during EAE induction. Furthermore, the over-expression of SRG3 may lead to reductions in Th1 and Th17 infiltration into the CNS via an increase in CNS-derived IL4 production and via the alternative activation of macrophages/microglia.

It has been demonstrated that Treg cells play a dominant role in the suppression of EAE pathogenesis and that the depletion of Treg cells results in accelerated and more severe EAE in mice [21, 35–37]. In this study, we demonstrated that the increased frequency of Treg cells in the spinal cord of β-actin-SRG3 Tg mice is associated with the attenuation of inflammation and disease severity. In addition, the relatively low frequency of Foxp3^+^ Treg cells in the spinal cord of CD2-SRG3 Tg mice during EAE induction might be due to enhanced inflammatory immune responses because pro-inflammatory cytokines such as IFNγ, TNFα, and IL12 have been reported to inhibit the TGFβ-induced generation of Treg cells [38]. Previously, it has been reported that Treg cells efficiently induce the differentiation of macrophages towards the M2 phenotype but that IL10/TGFβ-induced M2 macrophages facilitate the differentiation of naive T cells into Treg cells and IL10-producing M2 macrophages induce the recruitment of Treg cells into inflammatory lesion of CNS [39, 40]. Thus, cooperative activities of Treg cells and M2 macrophages might explain the completely opposing outcomes with respect to disease severity between CD2-SRG3 and β-actin-SRG3 Tg mice during EAE pathogenesis. Moreover, although TGFβ is necessary for Foxp3^+^ Treg differentiation, both CD2-SRG3 and β-actin-SRG3 Tg mice showed no significant difference in TGFβ expression of splenic DCs and macrophages compared with WT mice (S8 Fig).

Finally, it has previously been reported that innate immune cells such as basophils, mast cells, eosinophils, and NKT cells can produce IL4, leading to the initiation of Th2 immune responses [41, 42]. We therefore investigated whether these innate immune cells significantly induce a shift towards a Th2 immune response by more abundantly producing IL4 in β-actin-SRG3 Tg mice than in control mice during EAE development. However, IL4 expression was not detectable in any of the innate immune cell types in EAE-induced β-actin-SRG3 Tg mice. This result might be due to the suppression of IL4 production by a Th1-dominant immune response in EAE (S9 Fig). Nevertheless, we observed that basophils and mast cells in the spleen of β-actin SRG3 Tg mice respond to IL3 stimulation *in vitro*, resulting in a significant increase in both cell number and IL4 production. Because a previous study showed that the elevation of the basophil number affects spontaneous Th2 polarization in uninfected mice [43], it is possible that the increase in the number of basophils and mast cells in β-actin-SRG3 Tg mice influences the Th1/Th2 balance. Furthermore, two studies have recently reported that SRG3 directly interacts with another SWI/SNF subunit, BRG1, which is required for the expression of IL3, a critical growth factor that induces the differentiation of basophils and mast cells from bone marrow cells [44]. These reports suggest that the significant increase in both basophils and mast cells in β-actin-SRG3, but not CD2-SRG3, Tg mice was attributable to the SRG3 over-expression-mediated induction of high levels of IL3 production, which was responsible for an increase in Th2 innate immune cells.

In summary, our results demonstrate that β-actin promoter-driven SRG3 over-expression in DCs and macrophages modulates EAE pathogenesis. It has been reported that the induction of immunological tolerance against auto-antigens such as MBP largely depend on the administration route of antigens and type of antigen-pulsed APC [45, 46]. From the aspects of clinical application, enhancement of SRG3 expression in APCs derived from patients by either retroviral gene delivery or chemical treatment can provide another potential way to generate APCs to modulate Th1/Th17-biased immune responses in multiple sclerosis [47]. Moreover, because our previous study has shown that the modulation of T helper cell differentiation affects the pathogenesis of allergic diseases such as atopic dermatitis (AD), which is characterized by the predominant production of Th2 cells [48], the alteration of the Th1/Th2 balance by SRG3 expression may represent a therapeutic strategy to protect against the onset of allergic diseases.

## Supporting Information

S1 Fig SRG3 over-expression driven by the β-actin promoter reduced cytokine production in DCs following LPS stimulation. WT, β-actin-SRG3 Tg, and CD2-SRG3 Tg B6 mice were i.p. injected with LPS (2 μg) or vehicle. Sixteen hrs later, intracellular TNFα, IL12p40, iNOS, and IL10 production were assessed in splenic CD11c^+^ DCs by flow cytometric analysis. Representative FACS plots are shown (n = 4).
(PDF)

S2 Fig SRG3 over-expression driven by the β-actin promoter promotes the phenotypic shift of macrophages from M1 to M2 in response to LPS. (Figs A and B) Peritoneal macrophages isolated from both WT and β-actin-SRG3 Tg B6 mice were primed for 5 hrs with either IFNγ (20 ng/ml) or IL4 (20 ng/ml). IFNγ- and IL4-primed macrophages were stimulated with either vehicle or LPS (40, 200, or 1000 ng/ml). Twelves hrs later, IL12p40 and IL10 expression were analyzed in IFNγ- and IL4-primed macrophages respectively by flow cytometric analysis. The means ± SD are shown in the graphs (n = 3; *P<0.05, **P<0.01). (Fig C) WT, β-actin-SRG3 Tg and CD2-SRG3 Tg B6 mice were i.p. injected with LPS (2 μg) or vehicle. Sixteen hrs later, intracellular TNFα, IL12p40, iNOS, arginase-1 and IL10 production and the surface expression of Dectin-1 and MR1 in macrophages (CD11c^-^CD11b^+^F4/80^+^) were assessed by flow cytometric analysis. Representative FACS plots are shown (n = 4).
(PDF)

S3 Fig Phenotypic characterization of splenic mast cells and basophils. (Fig A) The frequencies of both mast cells (FcεRI^+^CD200R^-^CD3^-^B220^-^) and basophils (FcεRI^+^CD200R^+^CD3^-^B220^-^) in the spleen from WT mice were plotted. Representative data are shown (n = 3). (Fig B) The surface expressions of c-kit and DX5 in splenic basophils (FcεRI^+^CD200R^+^CD3^-^B220^-^) and mast cells (FcεRI^+^CD200R^-^CD3^-^B220^-^) from WT mice were determined by flow cytometry. One of representative data are shown (n = 3).
(PDF)

S4 Fig β-actin-SRG3 and CD2-SRG3 Tg mice showed no significant difference in cell number and IL4 production of eosinophils and NKT cells. Splenocytes were isolated from the spleens of WT, β-actin-SRG3 Tg, and CD2-SRG3 Tg B6 mice at the age of 8 weeks. (Figs A and B) The frequencies of both eosinophils (Siglec-F^+^CD3^-^CD19^-^) and NKT cells (NK1.1^+^CD3^+^) in the spleen were plotted. (Fig C) The absolute cell numbers of both eosinophils and NKT cells were determined. The means ± SD are shown (n = 3). (Fig D) Splenocytes from WT, β-actin-SRG3 Tg, and CD2-SRG3 Tg B6 mice at the age of 8 weeks were cultured in the presence of recombinant mIL3 (20 ng/ml) for 24 hrs. The percentages of IL4-producing cells among both eosinophils and NKT cells were analyzed via flow cytometry. The mean values ± SD are shown (n = 3). (Fig E) Splenocytes were prepared from WT, β-actin-SRG3 Tg, and CD2-SRG3 Tg B6 mice at 8 weeks of age. The absolute cell numbers of both IL4^+^ eosinophils and IL4^+^ NKT cells were determined. The means ± SD are shown (n = 3).
(PDF)

S5 Fig During EAE development, SRG3 over-expression driven by the CD2 promoter enhances Th1 and Th17 differentiation, whereas SRG3 over-expression driven by the β-actin promoter increases Th2 differentiation but decreases Th1 and Th17 differentiation. (Fig A) Both MBP TCR Tg B10.PL mice and CD2-SRG3/MBP TCR double Tg B10.PL mice or (Fig B) both MBP TCR Tg B10.PL mice and β-actin-SRG3/MBP TCR double Tg B10.PL mice were either non-immunized or s.c. immunized with the MBP-Ac1-11 peptide in CFA. (Figs A and B) CD4^+^ splenocytes purified from the four groups were activated with plate-bound anti-CD3 (10 μg/ml) and anti-CD28 (1 μg/ml) mAbs for 16 hrs and subsequently stimulated with PMA/ionomycin for 2 hrs in the presence of brefeldin A (10 μg/ml). The intracellular expression of IFNγ, IL17, IL4, and IL10 was evaluated by flow cytometric analysis. Representative FACS plots are shown (n = 5).
(PDF)

S6 Fig Comparison of Th1/Th2 and Th17/Treg ratios in the spleen and spinal cord between CD2-SRG3/MBP TCR double Tg mice and β-actin-SRG3/MBP TCR double Tg mice. Splenocytes (Figs A and B) and spinal cord-derived mononuclear cells (Fig B) were prepared from MBP TCR Tg B10.PL, CD2-SRG3/MBP TCR double Tg B10.PL, and β-acin-SRG3/MBP TCR double Tg B10.PL mice immunized with MBP to induce EAE. Th1/Th2 (Fig A) and Th17/Treg (Fig B) ratios of CD4^+^ T cells were evaluated in the spleen by flow cytometric analysis. The mean values ± SD are shown (n = 5; *P<0.05).
(PDF)

S7 Fig Comparison of M1/M2 ratio in the spleen and spinal cord between CD2-SRG3/MBP TCR double Tg mice and β-actin-SRG3/MBP TCR double Tg mice. Splenocytes and spinal cord-derived mononuclear cells were prepared from MBP TCR Tg B10.PL, CD2-SRG3/MBP TCR double Tg B10.PL, and β-acin-SRG3/MBP TCR double Tg B10.PL mice immunized with MBP to induce EAE. (Fig A) The numbers of total cells, M1 macrophages, and M2 macrophages infiltrated into the spinal cord were evaluated by flow cytometric analysis. The mean values ± SD are shown (n = 5; *P<0.05, **P<0.01). (Fig B) The M1/M2 ratio in MNCs from the spinal cord was also evaluated by flow cytometric analysis. The mean values ± SD are shown (n = 5; *P<0.05).
(PDF)

S8 Fig β-actin-SRG3 Tg and CD2-SRG3 Tg mice showed no significant difference in TGFβ production of DCs and macrophages in response to LPS stimulation. (Figs A and B) WT, β-actin-SRG3 Tg, and CD2-SRG3 Tg mice were i.p. injected with LPS (2 μg) or vehicle. Sixteen hrs later, intracellular TGFβ production was assessed in (Fig A) splenic CD11c^+^ DCs and (Fig B) macrophages (CD11c^-^CD11b^+^F4/80^+^) via flow cytometry. The means ± SD are shown in the graphs (n = 3; *P<0.05).
(PDF)

S9 Fig IL4 expression was not detectable in any of the innate immune cell types in both EAE-induced CD2-SRG3 Tg and β-actin-SRG3 Tg mice. (Figs A and B) Both MBP TCR Tg B10.PL mice and CD2-SRG3/MBP TCR double Tg B10.PL mice (left panel) or both MBP TCR Tg B10.PL mice and β-acin-SRG3/MBP TCR double Tg B10.PL mice (right panel) were either non-immunized or s.c. immunized with the MBP-Ac1-11 peptide in CFA. (Fig A) Subsequently, the frequencies of mast cells (FcεRI^+^CD200R^-^CD3^-^B220^-^), basophils (FcεRI^+^CD200R^+^CD3^-^B220^-^), eosinophils (Siglec-F^+^CD3^-^CD19^-^), and NKT cells (CD3^+^NK1.1^+^) were plotted. One of representative data are shown (n = 5). (Fig B) The percentages of IL4-producing cells among basophils, mast cells, eosinophils, and NKT cells were analyzed by flow cytometry. One of representative data are shown (n = 5).
(PDF)
