# Supplementary material for: Ubiquitous Over-Expression of Chromatin Remodeling Factor SRG3 Ameliorates the T Cell-Mediated Exacerbation of EAE by Modulating the Phenotypes of both Dendritic Cells and Macrophages
Source: PLoS One. 2015 Jul 6;10(7):e0132329. doi: 10.1371/journal.pone.0132329 (PMC4492541; doi:10.1371/journal.pone.0132329)
Supplement: S1 Fig — WT, β-actin-SRG3 Tg, and CD2-SRG3 Tg B6 mice were i.p. injected with LPS (2 μg) or vehicle. Sixteen hrs later, intracellular TNFα, IL12p40, iNOS, and IL10 production were assessed in splenic CD11c+ DCs by flow cytometric analysis. Representative FACS plots are shown (n = 4). (PDF) [file pone.0132329.s001.pdf]

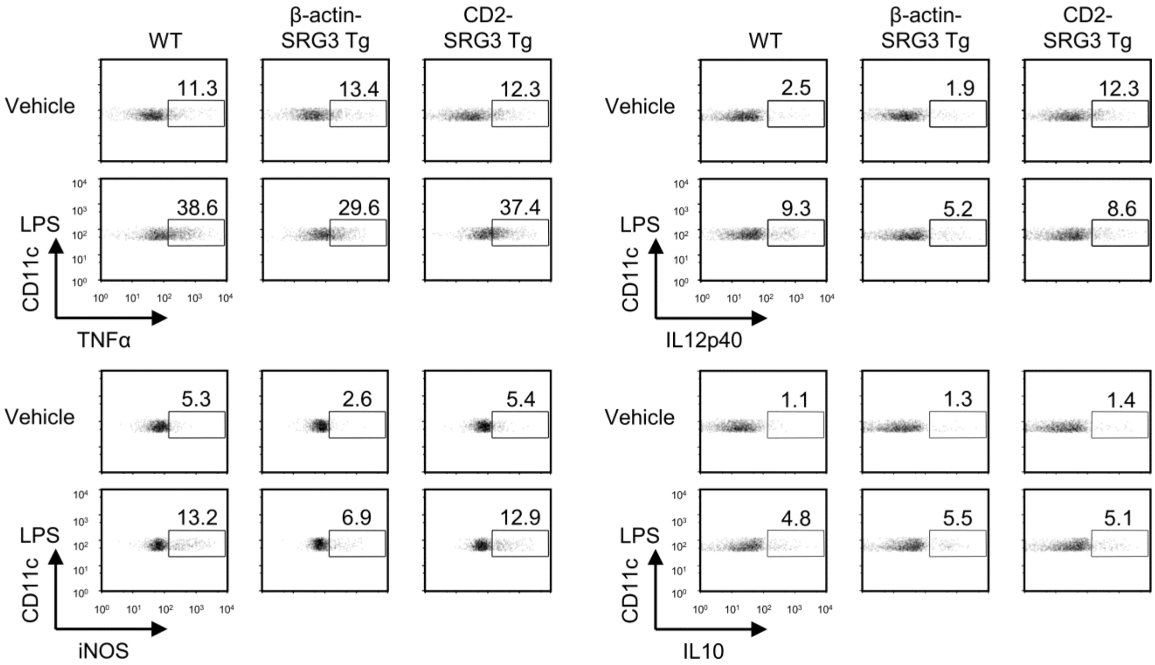

Figure S1. SRG3 over-expression driven by the β-actin promoter reduced cytokine production in DCs following LPS stimulation.

WT, β-actin-SRG3 Tg, and CD2-SRG3 Tg B6 mice were i.p. injected with LPS (2 μg) or vehicle. Sixteen hours later, intracellular TNFα, IL12p40, iNOS, and IL10 production were assessed in splenic CD11c^{+} DCs by flow cytometric analysis. Representative FACS plots are shown (n=4).
