# Supplementary material for: Ubiquitous Over-Expression of Chromatin Remodeling Factor SRG3 Ameliorates the T Cell-Mediated Exacerbation of EAE by Modulating the Phenotypes of both Dendritic Cells and Macrophages
Source: PLoS One. 2015 Jul 6;10(7):e0132329. doi: 10.1371/journal.pone.0132329 (PMC4492541; doi:10.1371/journal.pone.0132329)
Supplement: S2 Fig — (Figs A and B) Peritoneal macrophages isolated from both WT and β-actin-SRG3 Tg B6 mice were primed for 5 hrs with either IFNγ (20 ng/ml) or IL4 (20 ng/ml). IFNγ- and IL4-primed macrophages were stimulated with either vehicle or LPS (40, 200, or 1000 ng/ml). Twelves hrs later, IL12p40 and IL10 expression were analyzed in IFNγ- and IL4-primed macrophages respectively by flow cytometric analysis. The means ± SD are shown in the graphs (n = 3; *P<0.05, **P<0.01). (Fig C) WT, β-actin-SRG3 Tg and CD2-SRG3 Tg B6 mice were i.p. injected with LPS (2 μg) or vehicle. Sixteen hrs later, intracellular TNFα, IL12p40, iNOS, arginase-1 and IL10 production and the surface expression of Dectin-1 and MR1 in macrophages (CD11c-CD11b+F4/80+) were assessed by flow cytometric analysis. Representative FACS plots are shown (n = 4). (PDF) [file pone.0132329.s002.pdf]

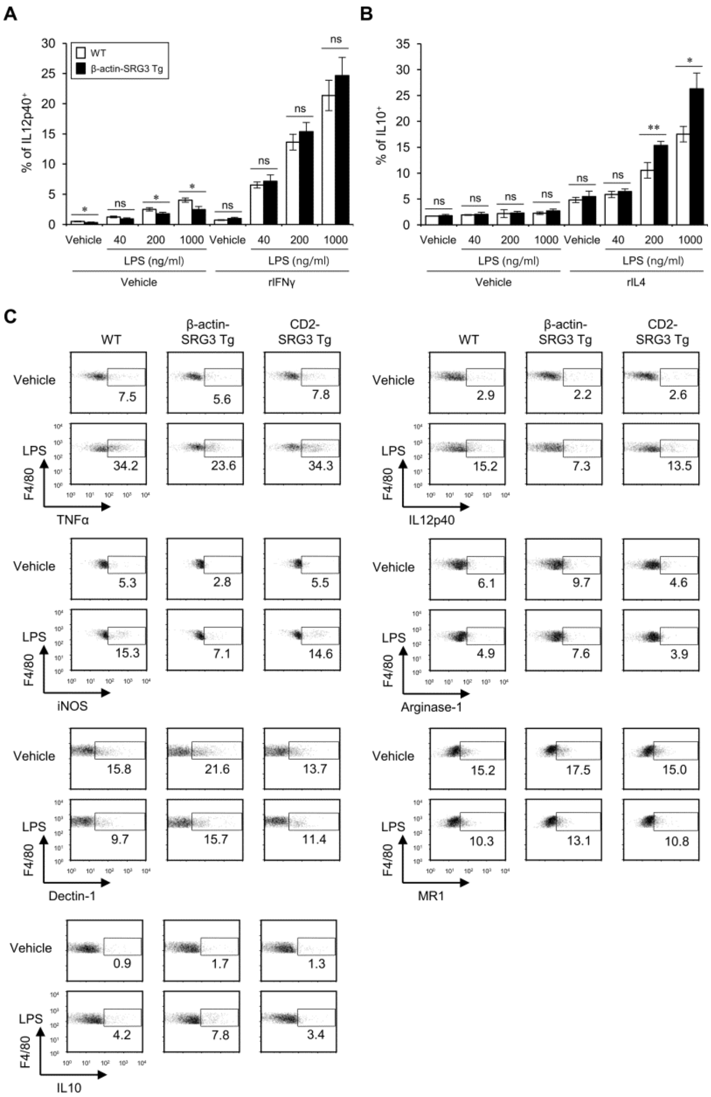

Figure S2. SRG3 over-expression driven by the β-actin promoter promotes the phenotypic shift of macrophages from M1 to M2 in response to LPS.

(A-B) Peritoneal macrophages isolated from both WT and β-actin-SRG3 Tg B6 mice were primed for 5 hrs with either IFNγ (20 ng/ml) or IL4 (20 ng/ml). IFNγ- and IL4-primed macrophages were stimulated with either vehicle or LPS (40, 200, or 1000 ng/ml). Twelves hrs later, IL12p40 and IL10 expression were analyzed in IFNγ- and IL4-primed macrophages respectively by flow cytometric analysis. The means ± SD are shown in the graphs (n=3; *P<0.05, **P<0.01). (C) WT, β-actin-SRG3 Tg and CD2-SRG3 Tg B6 mice were i.p. injected with LPS (2 μg) or vehicle. Sixteen hours later, intracellular TNFα, IL12p40, iNOS, arginase-1 and IL10 production and the surface expression of Dectin-1 and MR1 in macrophages ($CD11c^{-}CD11b^{+}F4/80^{+}$) were assessed by flow cytometric analysis. Representative FACS plots are shown (n=4).
