# Supplementary material for: Ubiquitous Over-Expression of Chromatin Remodeling Factor SRG3 Ameliorates the T Cell-Mediated Exacerbation of EAE by Modulating the Phenotypes of both Dendritic Cells and Macrophages
Source: PLoS One. 2015 Jul 6;10(7):e0132329. doi: 10.1371/journal.pone.0132329 (PMC4492541; doi:10.1371/journal.pone.0132329)
Supplement: S3 Fig — (Fig A) The frequencies of both mast cells (FcεRI+CD200R-CD3-B220-) and basophils (FcεRI+CD200R+CD3-B220-) in the spleen from WT mice were plotted. Representative data are shown (n = 3). (Fig B) The surface expressions of c-kit and DX5 in splenic basophils (FcεRI+CD200R+CD3-B220-) and mast cells (FcεRI+CD200R-CD3-B220-) from WT mice were determined by flow cytometry. One of representative data are shown (n = 3). (PDF) [file pone.0132329.s003.pdf]

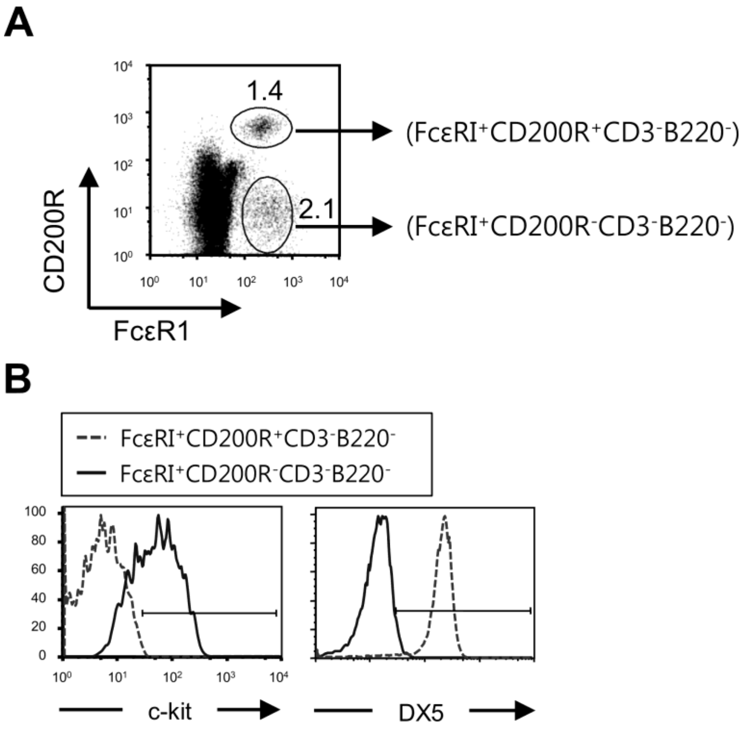

Figure S3. Phenotypic characterization of splenic mast cells and basophils

(A) The frequencies of both mast cells (FcεRI$^+$CD200R$^-$CD3$^-$B220$^-$) and basophils (FcεRI$^+$CD200R$^+$CD3$^-$B220$^-$) in the spleen from WT mice were plotted. Representative data are shown (n=3). (B) The surface expressions of c-kit and DX5 in splenic basophils (FcεRI$^+$CD200R$^+$CD3$^-$B220$^-$) and mast cells (FcεRI$^+$CD200R$^-$CD3$^-$B220$^-$) from WT mice were determined by flow cytometry. One of representative data are shown (n=3).
