# Supplementary material for: Ubiquitous Over-Expression of Chromatin Remodeling Factor SRG3 Ameliorates the T Cell-Mediated Exacerbation of EAE by Modulating the Phenotypes of both Dendritic Cells and Macrophages
Source: PLoS One. 2015 Jul 6;10(7):e0132329. doi: 10.1371/journal.pone.0132329 (PMC4492541; doi:10.1371/journal.pone.0132329)
Supplement: S4 Fig — Splenocytes were isolated from the spleens of WT, β-actin-SRG3 Tg, and CD2-SRG3 Tg B6 mice at the age of 8 weeks. (Figs A and B) The frequencies of both eosinophils (Siglec-F+CD3-CD19-) and NKT cells (NK1.1+CD3+) in the spleen were plotted. (Fig C) The absolute cell numbers of both eosinophils and NKT cells were determined. The means ± SD are shown (n = 3). (Fig D) Splenocytes from WT, β-actin-SRG3 Tg, and CD2-SRG3 Tg B6 mice at the age of 8 weeks were cultured in the presence of recombinant mIL3 (20 ng/ml) for 24 hrs. The percentages of IL4-producing cells among both eosinophils and NKT cells were analyzed via flow cytometry. The mean values ± SD are shown (n = 3). (Fig E) Splenocytes were prepared from WT, β-actin-SRG3 Tg, and CD2-SRG3 Tg B6 mice at 8 weeks of age. The absolute cell numbers of both IL4+ eosinophils and IL4+ NKT cells were determined. The means ± SD are shown (n = 3). (PDF) [file pone.0132329.s004.pdf]

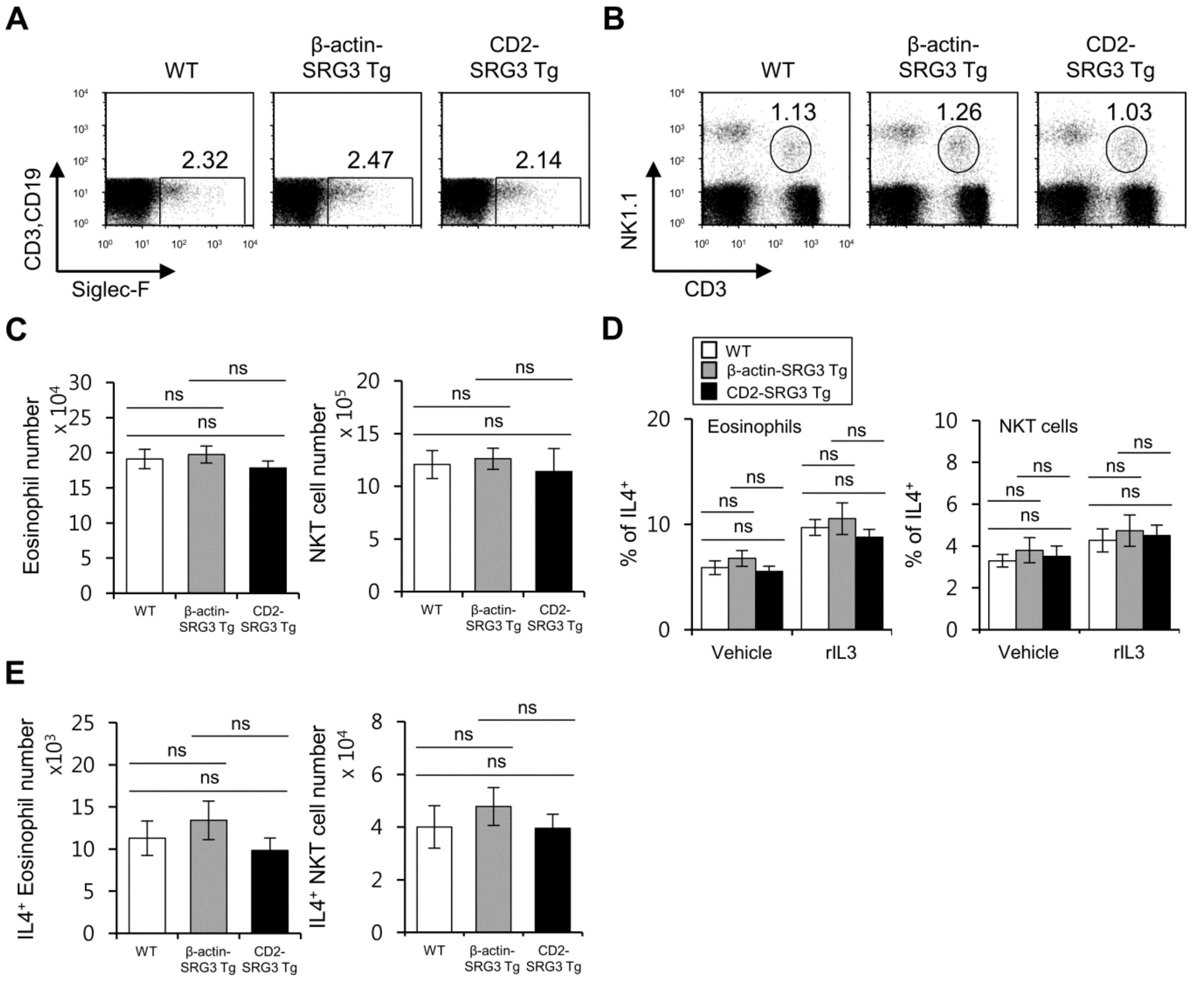

Figure S4. β-actin-SRG3 and CD2-SRG3 Tg mice showed no significant difference in cell number and IL4 production of eosinophils and NKT cells.

Splenocytes were isolated from the spleens of WT, β-actin-SRG3 Tg, and CD2-SRG3 Tg B6 mice at the age of 8 weeks. (A-B) The frequencies of both eosinophils (Siglec-$F^+CD3^-CD19^-$) and NKT cells ($NK1.1^+CD3^+$) in the spleen were plotted. (C) The absolute cell numbers of both eosinophils and NKT cells were determined. The means ± SD are shown (n=3). (D) Splenocytes from WT, β-actin-SRG3 Tg, and CD2-SRG3 Tg B6 mice at the age of 8 weeks were cultured in the presence of recombinant mIL3 (20 ng/ml) for 24 hrs. The percentages of IL4-producing cells among both eosinophils and NKT cells were analyzed via flow cytometry. The mean values ± SD are shown (n=3). (E) Splenocytes were prepared from WT, β-actin-SRG3 Tg, and CD2-SRG3 Tg B6 mice at 8 weeks of age. The absolute cell numbers of both $IL4^+$ eosinophils and $IL4^+$ NKT cells were determined. The means ± SD are shown (n=3).
