# Supplementary material for: Ubiquitous Over-Expression of Chromatin Remodeling Factor SRG3 Ameliorates the T Cell-Mediated Exacerbation of EAE by Modulating the Phenotypes of both Dendritic Cells and Macrophages
Source: PLoS One. 2015 Jul 6;10(7):e0132329. doi: 10.1371/journal.pone.0132329 (PMC4492541; doi:10.1371/journal.pone.0132329)
Supplement: S5 Fig — (Fig A) Both MBP TCR Tg B10.PL mice and CD2-SRG3/MBP TCR double Tg B10.PL mice or (Fig B) both MBP TCR Tg B10.PL mice and β-actin-SRG3/MBP TCR double Tg B10.PL mice were either non-immunized or s.c. immunized with the MBP-Ac1-11 peptide in CFA. (Figs A and B) CD4+ splenocytes purified from the four groups were activated with plate-bound anti-CD3 (10 μg/ml) and anti-CD28 (1 μg/ml) mAbs for 16 hrs and subsequently stimulated with PMA/ionomycin for 2 hrs in the presence of brefeldin A (10 μg/ml). The intracellular expression of IFNγ, IL17, IL4, and IL10 was evaluated by flow cytometric analysis. Representative FACS plots are shown (n = 5). (PDF) [file pone.0132329.s005.pdf]

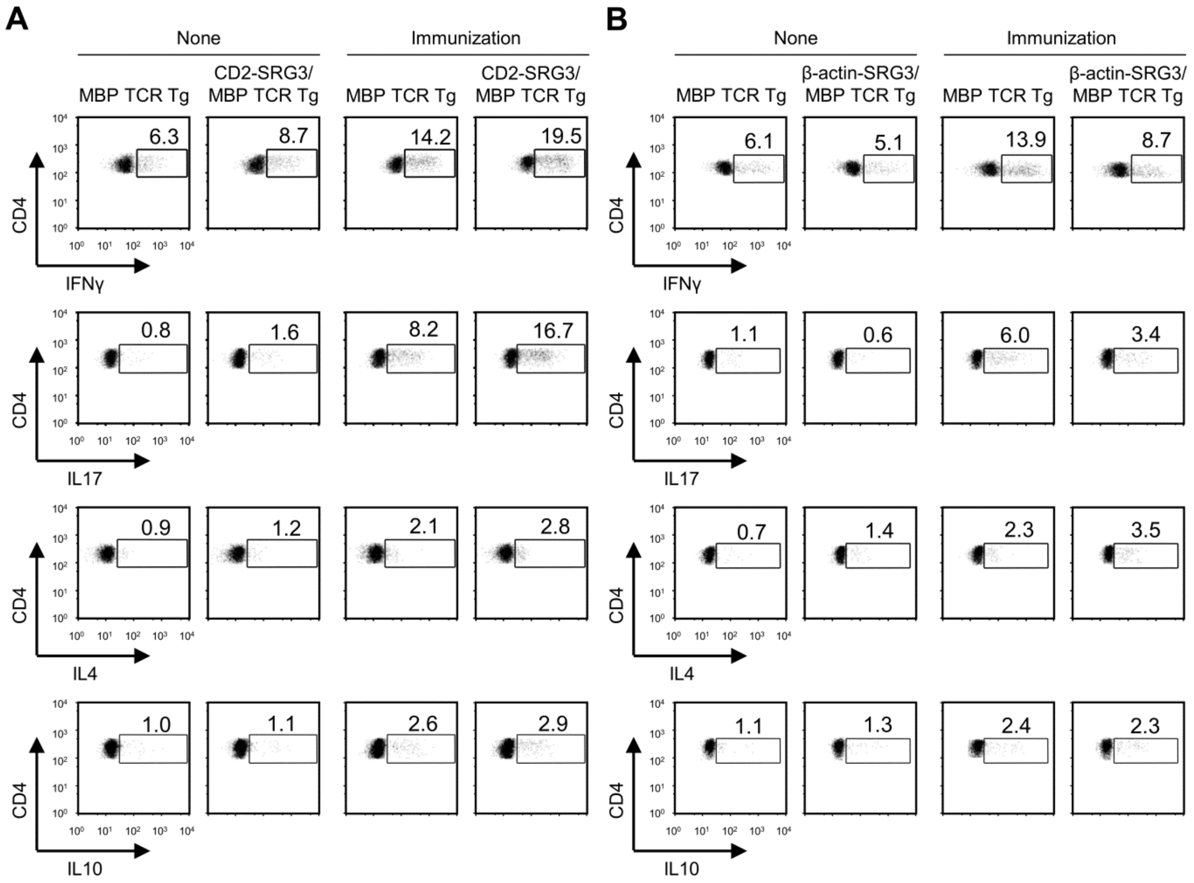

Figure S5. During EAE development, SRG3 over-expression driven by the CD2 promoter enhances Th1 and Th17 differentiation, whereas SRG3 over-expression driven by the β-actin promoter increases Th2 differentiation but decreases Th1 and Th17 differentiation.

(A) Both MBP TCR Tg B10.PL mice and CD2-SRG3/MBP TCR double Tg B10.PL mice or (B) both MBP TCR Tg B10.PL mice and β-actin-SRG3/MBP TCR double Tg B10.PL mice were either non-immunized or s.c. immunized with the MBP-Ac1-11 peptide in CFA. (A-B) $CD4^+$ splenocytes purified from the four groups were activated with plate-bound anti-CD3 (10 μg/ml) and anti-CD28 (1 μg/ml) mAbs for 16 hrs and subsequently stimulated with PMA/ionomycin for 2 hrs in the presence of brefeldin A (10 μg/ml). The intracellular expression of IFNγ, IL17, IL4, and IL10 was evaluated by flow cytometric analysis. Representative FACS plots are shown (n=5).
