# Supplementary material for: Ubiquitous Over-Expression of Chromatin Remodeling Factor SRG3 Ameliorates the T Cell-Mediated Exacerbation of EAE by Modulating the Phenotypes of both Dendritic Cells and Macrophages
Source: PLoS One. 2015 Jul 6;10(7):e0132329. doi: 10.1371/journal.pone.0132329 (PMC4492541; doi:10.1371/journal.pone.0132329)
Supplement: S6 Fig — Splenocytes (Figs A and B) and spinal cord-derived mononuclear cells (Fig B) were prepared from MBP TCR Tg B10.PL, CD2-SRG3/MBP TCR double Tg B10.PL, and β-acin-SRG3/MBP TCR double Tg B10.PL mice immunized with MBP to induce EAE. Th1/Th2 (Fig A) and Th17/Treg (Fig B) ratios of CD4+ T cells were evaluated in the spleen by flow cytometric analysis. The mean values ± SD are shown (n = 5; *P<0.05). (PDF) [file pone.0132329.s006.pdf]

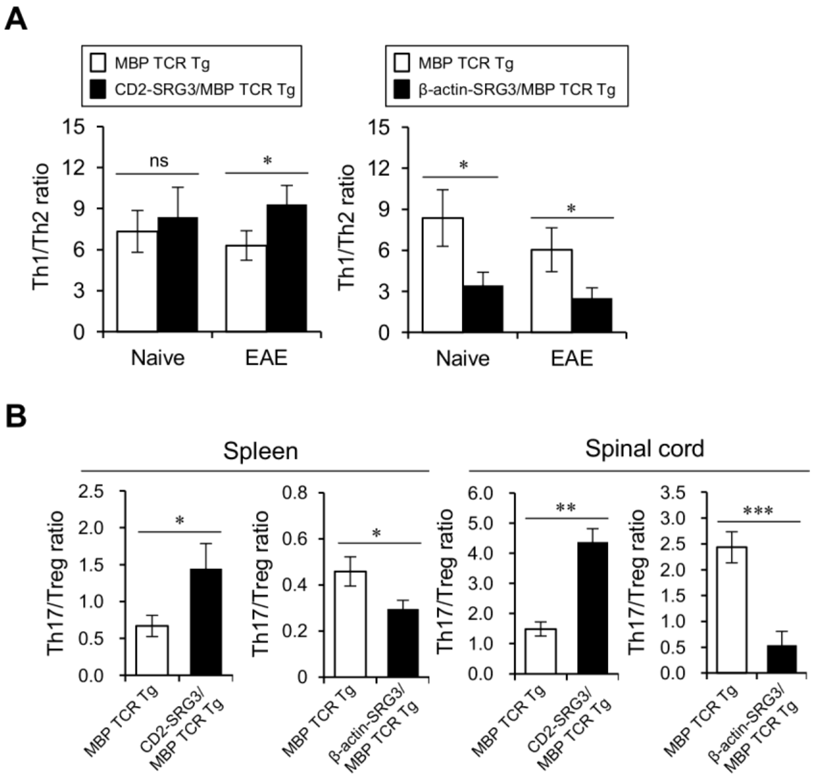

Figure S6. Comparison of Th1/Th2 and Th17/Treg ratios in the spleen and spinal cord between CD2-SRG3/MBP TCR double Tg mice and β-actin-SRG3/MBP TCR double Tg mice.

Splenocytes (A-B) and spinal cord-derived mononuclear cells (B) were prepared from MBP TCR Tg B10.PL, CD2-SRG3/MBP TCR double Tg B10.PL, and β-acin-SRG3/MBP TCR double Tg B10.PL mice immunized with MBP to induce EAE. Th1/Th2 (A) and Th17/Treg (B) ratios of $CD4^+$ T cells were evaluated in the spleen by flow cytometric analysis. The mean values ± SD are shown (n=5; *P<0.05).
