# Supplementary material for: Ubiquitous Over-Expression of Chromatin Remodeling Factor SRG3 Ameliorates the T Cell-Mediated Exacerbation of EAE by Modulating the Phenotypes of both Dendritic Cells and Macrophages
Source: PLoS One. 2015 Jul 6;10(7):e0132329. doi: 10.1371/journal.pone.0132329 (PMC4492541; doi:10.1371/journal.pone.0132329)
Supplement: S7 Fig — Splenocytes and spinal cord-derived mononuclear cells were prepared from MBP TCR Tg B10.PL, CD2-SRG3/MBP TCR double Tg B10.PL, and β-acin-SRG3/MBP TCR double Tg B10.PL mice immunized with MBP to induce EAE. (Fig A) The numbers of total cells, M1 macrophages, and M2 macrophages infiltrated into the spinal cord were evaluated by flow cytometric analysis. The mean values ± SD are shown (n = 5; *P<0.05, **P<0.01). (Fig B) The M1/M2 ratio in MNCs from the spinal cord was also evaluated by flow cytometric analysis. The mean values ± SD are shown (n = 5; *P<0.05). (PDF) [file pone.0132329.s007.pdf]

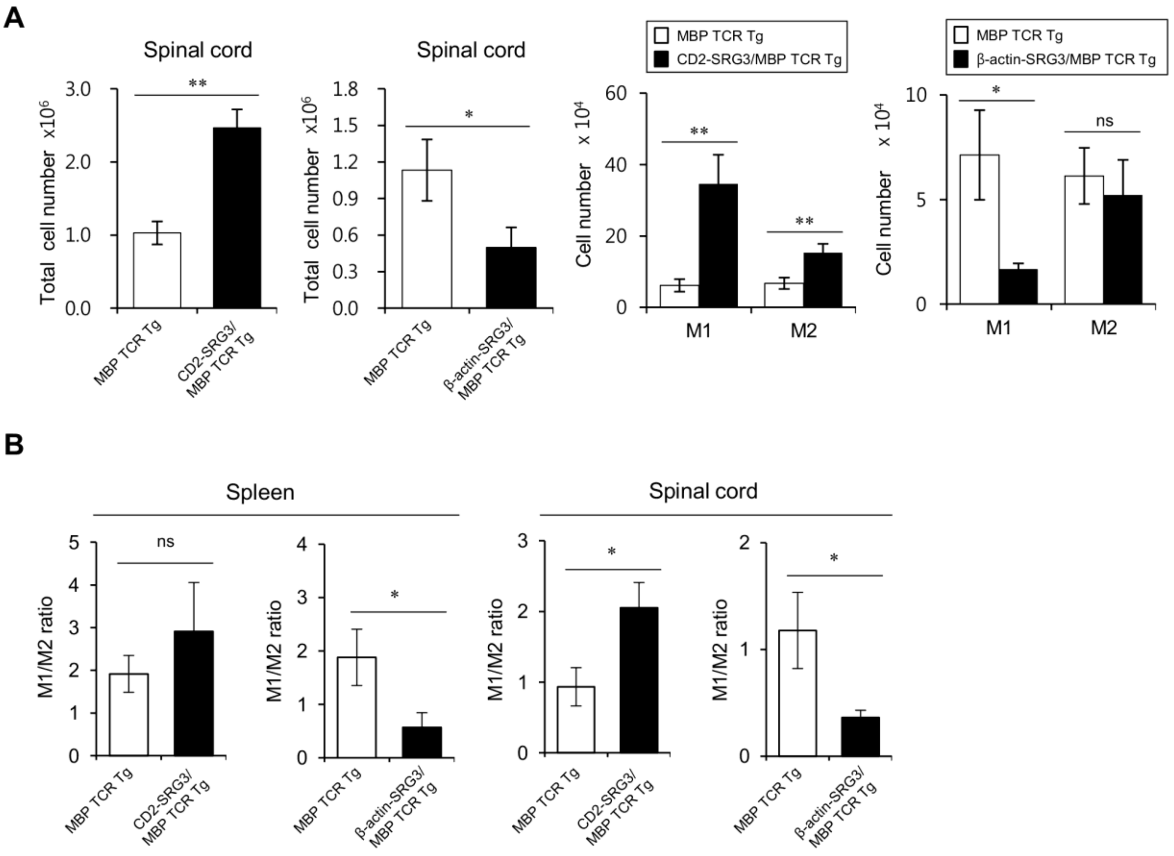

Figure S7. Comparison of M1/M2 ratio in the spleen and spinal cord between CD2-SRG3/MBP TCR double Tg mice and β-actin-SRG3/MBP TCR double Tg mice.

Splenocytes and spinal cord-derived mononuclear cells were prepared from MBP TCR Tg B10.PL, CD2-SRG3/MBP TCR double Tg B10.PL, and β-acin-SRG3/MBP TCR double Tg B10.PL mice immunized with MBP to induce EAE. (A) The numbers of total cells, M1 macrophages, and M2 macrophages infiltrated into the spinal cord were evaluated by flow cytometric analysis. The mean values ± SD are shown (n=5; *P<0.05, **P<0.01). (B) The M1/M2 ratio in MNCs from the spinal cord was also evaluated by flow cytometric analysis. The mean values ± SD are shown (n=5; *P<0.05).
