# Supplementary material for: Ubiquitous Over-Expression of Chromatin Remodeling Factor SRG3 Ameliorates the T Cell-Mediated Exacerbation of EAE by Modulating the Phenotypes of both Dendritic Cells and Macrophages
Source: PLoS One. 2015 Jul 6;10(7):e0132329. doi: 10.1371/journal.pone.0132329 (PMC4492541; doi:10.1371/journal.pone.0132329)
Supplement: S8 Fig — (Figs A and B) WT, β-actin-SRG3 Tg, and CD2-SRG3 Tg mice were i.p. injected with LPS (2 μg) or vehicle. Sixteen hrs later, intracellular TGFβ production was assessed in (Fig A) splenic CD11c+ DCs and (Fig B) macrophages (CD11c-CD11b+F4/80+) via flow cytometry. The means ± SD are shown in the graphs (n = 3; *P<0.05). (PDF) [file pone.0132329.s008.pdf]

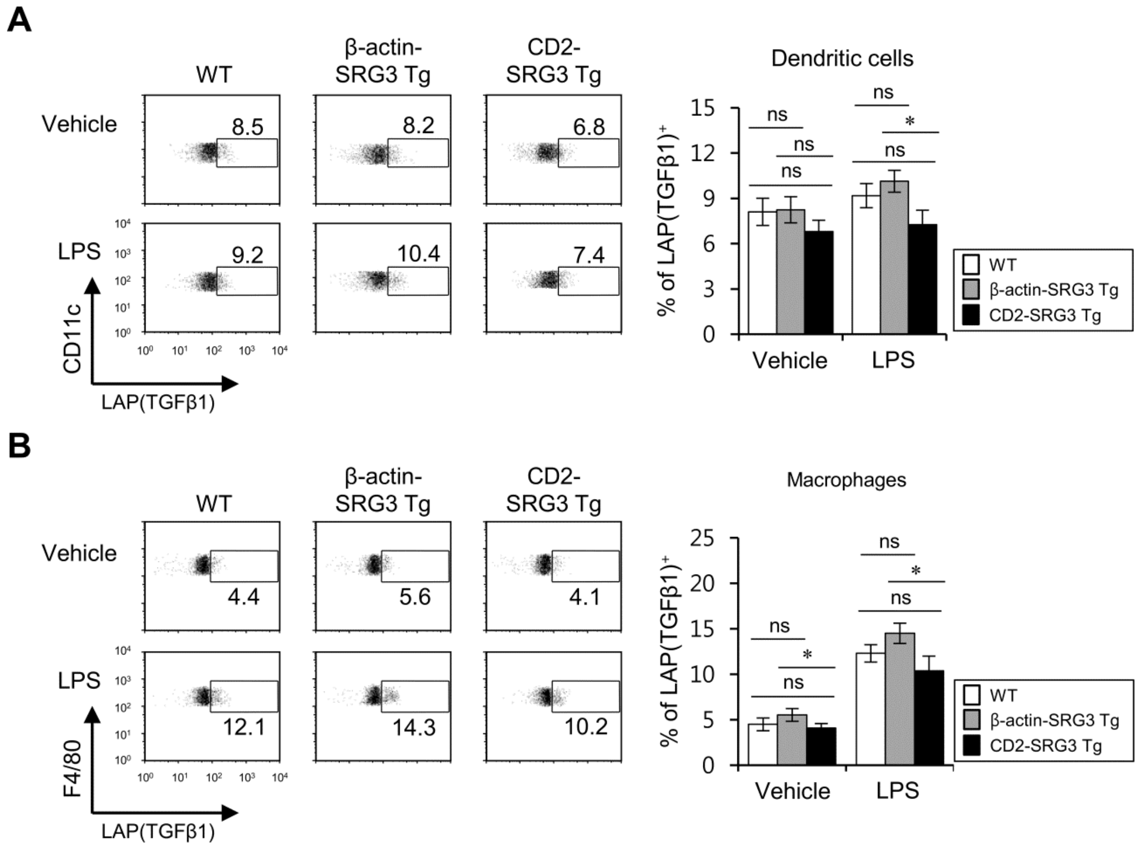

Figure S8. β-actin-SRG3 Tg and CD2-SRG3 Tg mice showed no significant difference in TGFβ production of DCs and macrophages in response to LPS stimulation.

(A-B) WT, β-actin-SRG3 Tg, and CD2-SRG3 Tg mice were i.p. injected with LPS (2 μg) or vehicle. Sixteen hrs later, intracellular TGFβ production was assessed in (A) splenic $CD11c^{+}$ DCs and (B) macrophages ($CD11c^{-}$ $CD11b^{+}F4/80^{+}$) via flow cytometry. The means ± SD are shown in the graphs (n=3; *P<0.05).
