# Supplementary material for: Ubiquitous Over-Expression of Chromatin Remodeling Factor SRG3 Ameliorates the T Cell-Mediated Exacerbation of EAE by Modulating the Phenotypes of both Dendritic Cells and Macrophages
Source: PLoS One. 2015 Jul 6;10(7):e0132329. doi: 10.1371/journal.pone.0132329 (PMC4492541; doi:10.1371/journal.pone.0132329)
Supplement: S9 Fig — (Figs A and B) Both MBP TCR Tg B10.PL mice and CD2-SRG3/MBP TCR double Tg B10.PL mice (left panel) or both MBP TCR Tg B10.PL mice and β-acin-SRG3/MBP TCR double Tg B10.PL mice (right panel) were either non-immunized or s.c. immunized with the MBP-Ac1-11 peptide in CFA. (Fig A) Subsequently, the frequencies of mast cells (FcεRI+CD200R-CD3-B220-), basophils (FcεRI+CD200R+CD3-B220-), eosinophils (Siglec-F+CD3-CD19-), and NKT cells (CD3+NK1.1+) were plotted. One of representative data are shown (n = 5). (Fig B) The percentages of IL4-producing cells among basophils, mast cells, eosinophils, and NKT cells were analyzed by flow cytometry. One of representative data are shown (n = 5). (PDF) [file pone.0132329.s009.pdf]

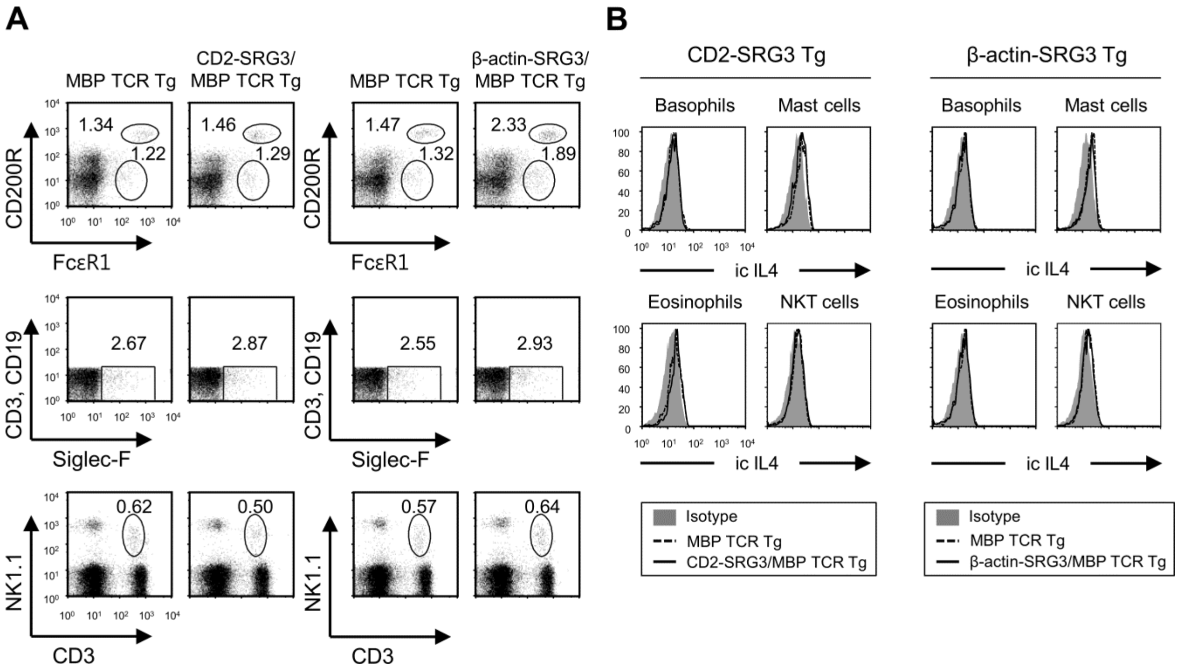

Figure S9. IL4 expression was not detectable in any of the innate immune cell types in both EAE-induced CD2-SRG3 Tg and β-actin-SRG3 Tg mice.

(A and B) Both MBP TCR Tg B10.PL mice and CD2-SRG3/MBP TCR double Tg B10.PL mice (left panel) or both MBP TCR Tg B10.PL mice and β-acin-SRG3/MBP TCR double Tg B10.PL mice (right panel) were either non-immunized or s.c. immunized with the MBP-Ac1-11 peptide in CFA. (A) Subsequently, the frequencies of mast cells (FcεRI$^+$CD200R$^-$CD3$^-$B220$^-$), basophils (FcεRI$^+$CD200R$^+$CD3$^-$B220$^-$), eosinophils (Siglec-F$^+$CD3$^-$CD19$^-$), and NKT cells (CD3$^+$NK1.1$^+$) were plotted. One of representative data are shown (n=5). (B) The percentages of IL4-producing cells among basophils, mast cells, eosinophils, and NKT cells were analyzed by flow cytometry. One of representative data are shown (n=5).
